# Diversity, biofilm formation and antimicrobial susceptibility of aerobic heterotrophic bacteria isolated from cooling towers

**DOI:** 10.1007/s11274-026-05127-1

**Published:** 2026-07-14

**Authors:** Marcus Vinícius Dias-Souza, Andrea Lima Alves, Ubiana de Cássia Mourão Silva, Aline Daniela Lopes Júlio, Andréa Veiga, Sergio Pagnin, Vera Lúcia dos Santos

**Affiliations:** 1 Applied Microbiology Laboratory, Microbiology Department, Instituto de Ciências Biológicas, Universidade Federal de Minas Gerais, Belo Horizonte, C.P. 486, 31270-901 MG Brazil; 2 Petróleo Brasileiro S.A. Research and Development Center (CENPES), Cidade Universitária, Av. Horácio Macedo950, Ilha do Fundão - Rio de Janeiro, ZIP 21941-915 RJ Brazil

**Keywords:** Cooling towers, Corrosion, Microbial biofilm, Antimicrobial susceptibility

## Abstract

Cooling towers (CTw) are essential for industrial refrigeration but provide favourable conditions for microbial growth and biofilm formation, potentially facilitating the spread of antimicrobial-resistant bacteria (ARB). This study examines bacterial diversity, biofilm potential and antimicrobial susceptibility in two industrial CTw in Brazil using culture-based and metataxonomic (16 S rRNA gene amplicon sequencing) approaches. Fourteen bacterial genera and 22 species were identified, including *Bacillus* spp. (60% of isolates), *Acinetobacter* spp. (14%) and others like *Pseudomonas*,* Serratia*, and *Ochrobactrum*, many linked to biofilm formation and ARB. Metataxonomic analysis revealed a broader and more diverse microbial community, comprising families such as *Burkholderiaceae*,* Comamonadaceae*,* and Sphingomonadaceae*, which are known for their biofilm resilience. Pathogens including *Legionella* were also detected. Bacterial richness was higher in CTw 2, likely due to untreated industrial and domestic effluent inputs, whereas CTw 1, supplied with treated secondary industrial effluent, exhibited lower diversity. Functional predictions indicated genes associated with biofilm formation, quorum-sensing, motility and xenobiotic degradation. Antimicrobial susceptibility testing showed high resistance to β-lactams and nitrofurantoin, with meropenem being the most effective. Higher resistance rates in CTw 2 suggest selective pressure from industrial contaminants. These findings underscore the complex microbial ecology of CTw and the coexistence of cultivable and non-cultivable bacteria with biofilm-forming capacity and antimicrobial resistance traits in these systems.

**Supplementary Information:**

The online version contains supplementary material available at 10.1007/s11274-026-05127-1.

## Introduction

Cooling towers (CTw) are large-scale devices designed to dissipate heat from equipment and systems, ensuring the proper operation of industrial processes. The cooling mechanism typically involves heat transfer through the direct contact of hot water with atmospheric air (Deziani et al. 2017). The recirculation of water with organic matter combined with suitable pH and temperature levels creates an ideal environment for microbial growth and biofilm formation. Moreover, the atmospheric air and water supply are the primary pathways for microbial entry into CTw (Doğruöz et al. 2009; Liu et al. 2009), where microorganisms can exist either as planktonic (free-floating) cells or as sessile communities embedded in biofilms.

Biofilms are structured microbial communities that enhance microorganism survival under adverse conditions such as pH extremes, nutrient scarcity, antimicrobial agents and host immune responses (Hall-Stoodley et al. 2004). Compared to planktonic bacteria, sessile bacteria within biofilms exhibit differences in growth rate, the production of adhesive extracellular polymeric substances (EPS), and reduced susceptibility to antimicrobial compounds. In environmental settings and in biological organisms, biofilm communities are mostly of polymicrobial composition. Biofilm formation begins when planktonic cells attach to surfaces, form microcolonies and subsequently develop into mature, dynamic biofilms through specific adhesion and aggregation mechanisms (Liu et al. 2009; Hall-Stoodley et al. 2004).

Microbial biofilms are associated with several challenges in CTw, with biofouling being one of the most significant due to the economic burden related to maintenance (Bott 1998; Chien et al. 2013). Biofilm formation increases the risk of microbiologically induced corrosion, which accelerates biofouling on wet surfaces. This can lead to equipment inefficiency or even failure, as biofouling impedes heat transfer across contaminated surfaces and clogs hydraulic systems (Characklis et al. 1981). The extent and type of biofouling depend on factors such as water quality, the presence of organic and inorganic matter and the microbial species present in biofilms (Chien et al. 2013). Moreover, pathogenic and opportunistic microorganisms may be present in CTw and can be dispersed into the environment through aerosolized water, exposing nearby humans and animals to infectious agents (Ruíz et al. 2013). Infectious diseases linked to cooling towers, such as legionellosis outbreaks caused by *Legionella* bacteria, have been associated with CTw world-wide (Osawa et al. 2014; Reuters 2016; Tsao et al. 2019). Notably, in 2024, a community outbreak of Legionnaires’ disease was reported in New Hampshire, USA, with five confirmed cases linked to exposure to a cooling tower in Lincoln, prompting public health investigations and control measures (New Hampshire Department of Health and Human Services (NH DHHS, 2024). In 2025, a significant outbreak occurred in Harlem, New York City, resulting in over 110 confirmed cases and at least seven deaths, leading to extensive public health interventions, including disinfection of implicated cooling towers (New York City Department of Health (NYC DOH) 2025; CBS News 2025). These incidents underscore the persistent public health hazards posed by Legionella in cooling systems.

Several other microbial species have been detected in CTw, including *Legionella pneumophila*,* Naegleria fowleri*,* Hartmanella vermiformis*,* Burkholderia cepacia*,* Pseudomonas fluorescens*,* Pasteurella haemolytica*,* Aeromonas caviae and Pantoea agglomerans* (Ceyhan and Özdemir 2008; Pagnier et al. 2009). Beyond pathogenicity, CTw biofilms often harbor microorganisms implicated in microbiologically influenced corrosion (MIC), such as sulfate-reducing bacteria (SRB) from the genera *Desulfovibrio*,* Desulfomicrobium*,* Desulfonema* and *Desulfosarcina*, which promote metal sulfide deposition and corrosion processes (Rao and Mulky 2023; Li et al. 2024). Other corrosion-related groups include iron-oxidizing and manganese-oxidizing bacteria (*Gallionella* spp, *Leptothrix* spp.), acid-producing bacteria (*Acidithiobacillus* spp.) and even methanogenic archaea in low-oxygen niches (Beech and Sunner 2004; Li et al. 2024). Despite the recognized diversity of CTw microbiomes, most studies still describe microbial communities only at broad taxonomic levels, without detailed insights into functional roles in biofouling, corrosion, or health risks (Liu et al. 2009; Pagnier et al. 2009).

In recent years, several studies have focused on strategies for evaluating and controlling biological growth in cooling water. However, limited research has investigated the potential environmental and health risks associated with antibiotic resistance when discharging used cooling water into receiving water bodies. There is evidence suggesting that the chemical treatment of water for tower supply may influence microbial resistance to antimicrobial compounds. Treatment systems often create environments where microorganisms are exposed to sub-inhibitory concentrations of antimicrobial agents, promoting horizontal gene transfer and increasing bacterial resistance. This complicates bacterial control without exceeding standard biocide doses and transforms CTw into significant point sources of antimicrobial resistance genes (ARGs) released into the environment (Critchley and Bentham 2009; Dias-Souza et al. 2024; Alves Ruislan et al. 2024).

Given these concerns, this study aimed to characterize the bacterial communities present in two industrial-scale cooling towers (CTws) from a Brazilian industry using both culture-dependent and culture-independent approaches. Additionally, we evaluated the antibiotic resistance and biofilm formation phenotypes of the isolated bacterial strains. By combining cultivation-based methods with high-throughput sequencing of 16 S rRNA gene amplicons, we obtained a comprehensive overview of the microbial communities inhabiting these systems. Notably, several bacterial taxa identified in this study have not previously been reported in cooling towers, and many isolates exhibited reduced susceptibility to commonly used antimicrobial agents. Our findings highlight the need for further investigations into the quality of water used in CTw and the potential risks associated with the environmental dissemination of pathogenic or antibiotic-resistant bacteria and ARGs through aerosolized water and discharged cooling water.

## Materials and methods

### CTw system and water treatment

Aerobic total heterotrophic bacteria (THB) were isolated from water samples collected from two industrial-scale CTw systems at a Brazilian industrial plant. These systems utilize either treated recycled water (CTw 1) or clarified and chlorinated water (CTw 2) as makeup water. Operational and physicochemical parameters of the CTw during the experimental period are presented in Tables 1 and 2.

**Table 1:** Operational parameters monitored in the cooling tower

Parameter	CTw 1	CTw 2
Capacity (m^3^)	903	2160
Recirculation rate (m3/h)	2415.4	4732
Evaporation rate (m3/h)	35.8	97.9
Windage (m3/h)	2.42	4.73
Temperature of the water of return (^o^C)	38.5	38
Temperature of the basin of cold water (^o^C)	29.9	26
Cooling rate (^o^C)	8.6	12
Total liquid purge (m3/h)	11.87	30.88
Residence time (h)	76.1	69.9
Makeup water (m3/h)	47.7	128.8
Concentration cycles	4	4.2

* Values represent the averages obtained during the experimental period

**Table 2:** Physicochemical parameters monitored in the cooling tower

Parameter	CTw 1			CTw 2		
	Min	Avg	Max	Min	Avg	Max
Bicarbonate alkalinity (mg/L)	11.89	217.22	368.39	98.37	344.1	734.44
Al (mg/L)	0.01	1.48	4.2	0.01	0.48	0.12
NH_3_-N (mg/L)	0.17	1.84	2.04	0.34	1.25	9.82
Chlorides (mg/L Cl^−^)	139.52	415.55	623.15	125.1	444.43	1125.94
Residual-free chlorine (mg/L)	0.01	0.16	0.83	0.01	0.62	1.45
Chloramine (mg/L)	0.01	1.07	1.8	0.09	1.15	2.07
Conductivity (µS/cm)	710	2109.67	3150	1081	2288.46	3030
Color	10	123.07	150	2	85	150
Chemical Oxygen Demand	3.83	17.16	20.19	9.32	14.43	18.45
Hardness (mg/L of Ca^+ 2^)	10	115.87	212.85	116.48	259.95	433.44
Total hardness (mg/L CaCO_3_)	50	158	289.15	243.95	337.65	465.7
Total Fe (mg/L)	0.01	0.96	6.7	0.13	0.46	0.89
Soluble phosphate (ppm PO4^− 3^)	0	3.11	17.16	0.4	0.86	0.92
NO_3_-N (mg/L)	0	57.79	120	5	18.23	59
Oils and greases (mg/L)	0.06	0.74	3.1	0.4	0.6	0.8
pH (25 °C)	6	8.54	9.22	8.1	8.92	9.15
SiO_2_ (mg/L)	17.7	38.33	70.68	14.72	38.72	57.9
TSS (mg/L)	2.6	17.48	90.64	21	36.18	44
Sulfate (mg/L SO_4_^−2^)	14.83	209.64	475.86	12	263.84	371.4
TOC (mg/L)	16.79	60.88	175.5	10.59	18.22	23.61
Turbidity (NTU)	2.31	10.07	31.2	2.11	13.87	36.4
Soluble Zn (mg/L)	0.23	1.4	3.8	0.18	0.51	0.9
Concentration cycle (weighted average of Ca hardness and SiO_2_)	8.6	10.9	13.3	4.6	6.1	6.2

*Values are referent to observations during the experimental period

CTw 1 uses a secondary effluent generated from the Industrial Waste Treatment Plant, which undergoes treatment in a prototype reuse unit. The secondary treatment process involves two aerated lagoons and a system of rotating biological contactors. The water is initially chlorinated with sodium hypochlorite (NaOCl), followed by the addition of Actiflo, a high-efficiency clarifier, and then directed to sand filters to remove turbidity. After this step, another NaOCl dosage is applied, and the effluent is passed through activated carbon filters to remove organic waste. A third chlorine dosage is then administered before the water is directed to cartridge filters.

Subsequently, the water undergoes further treatment in the prototype reuse unit through reverse electrodialysis, which involves two pretreatment steps: (1) the removal of suspended solids via coagulation, flocculation, sedimentation, and sand filtration, and (2) the elimination of dissolved organic compounds through activated carbon filters. The treated effluent is then transferred to the tower supply tank, maintaining a free residual chlorine concentration of approximately 0.5 mg/L and chloramine levels between 1 and 1.5 mg/L.

CTw 2 is supplied with clarified and chlorinated water collected from an artificial lagoon in Minas Gerais. This water undergoes a pre-chlorination process to optimize coagulation and clarification. After the clarification stage, an additional chlorine dosage is applied to maintain free residual chlorine levels between 0.5 and 1.0 mg/L. Before being supplied to the tower, the water passes through sand filters to remove particulate matter.

### Enumeration and isolation of bacteria

A total of 36 glass slides (76 mm x 26 mm x 1.3 mm) were vertically inserted into plastic carriers and immersed in the water basins of both CTw to facilitate biofilm formation. These carriers were designed to securely hold the slides, preventing displacement due to water flow. Slides were collected at intervals of 7, 14, and 21 days using sterile containers (50 mL Falcon-type tubes) containing sterile saline solution (0.85% NaCl). Six slides were retrieved at each collection point.

Biofilm-associated cells were detached from the slide surfaces using an ultrasonic bath (three cycles of two minutes at 40 kHz) followed by vortexing for 60 s at maximum speed (Ruislan et al. 2004). Serial dilutions (10⁻¹ to 10⁻⁷) were prepared using sterile saline, and 100 µL aliquots were plated on plate count agar (PCA, Oxoid). Plates were incubated at 37 °C for 24–48 h, and colony-forming units (CFUs) were counted and expressed as CFUs per cm² of slide surface.

Additionally, 1 L water samples were collected directly from the CTw basins in sterile glass flasks for planktonic heterotrophic bacteria enumeration. Samples were homogenized immediately after collection, and serial decimal dilutions (10⁻¹ to 10⁻⁷) were prepared in sterile saline solution (0.85% NaCl). Aliquots (100 µL) of each dilution were spread onto PCA plates according to APHA (1998, Method 9215 C). Plates were incubated under the same conditions as the biofilm samples. CFUs for planktonic samples were expressed per mL. Colony morphotypes were characterized, and a total of 160 distinct morphotypes were identified based on colony color, surface texture, and margin characteristics.

### Bacterial community profile based on 16 S rRNA gene high-throughput sequencing

To investigate sessile bacterial communities, 16 S rRNA gene amplicon sequencing analysis was conducted on biofilm samples from 7-day (samples 7SA and 7SB) and 14-day (samples 14SA and 14SB) slides. Sessile cells detached from the slides were filtered through 0.22 μm membranes (Millipore, Germany) for DNA extraction using the PowerWater DNA Kit (MoBIO Laboratories). DNA quality and concentration were verified by agarose gel electrophoresis and Nanodrop™ 1000 (ThermoFisher).

V3-V4 regions of the 16 S rRNA gene were amplified using Illumina overhang-containing primers 341 F and 806R (Klindworth et al. 2013). PCR reaction was in a final volume of 25 µL containing 12.5 µL of PCR buffer (2x KAPA HiFi HotStart ReadyMix), 5 µM primers, and 30 ng of DNA. Amplifications comprised the following cycles: 95 °C/3 min for initial denaturation, followed by 30 cycles at 95 °C/30 s, 60 °C/30 s for annealing, and 72 °C/30 s for extension, with final extension at 72 °C/5 min. The amplicons were purified with AMPure XP beads (Beckman Coulter Genomics, USA) and ligated to an index sequence in a second PCR, containing 12.5 µL PCR buffer (2x KAPA HiFi HotStart ReadyMix), 3 µL of each Nextera index XT, 2.5 µL of the purified PCR product, and 7 µL of ultrapure water, in a final volume of 25 µL. The amplification was performed at 95 °C/3 min, followed by 8 cycles at 95 °C/30 s, 55 °C/30 s and 72 °C/30 s, with final extension at 72 °C/5 min. The new amplicons were purified again using AMPure XP beads and quantitated using Kapa KK4824 kit (Biosciences, USA). The size of the libraries was verified using Bioanalyzer DNA 1000 Assay (Agilent, USA). The libraries (2 × 300 bp) were sequenced in a MiSeq platform using MiSeq Reagent V3 kit (Illumina, USA).

Sequence analyses were done according to Pylro et al. (2014), briefly described next. Quality control was performed using Trimmomatic v.0.32 (Bolger et al. 2014), with an average Phred score of 15 and a four base sliding window. Sequences were truncated at 400 bp, and singletons and chimeras were removed using USEARCH (Edgar 2010). Taxonomic classification was performed with QIIME (Caporaso et al. 2010), based on Greengenes_13_08 database, with 97% similarity. Community profile and diversity analysis were performed with Phyloseq package (McMurdie and Holmes 2013) in R software (v4.2.1; R Core Team, 2022). Functional potential was estimated with PICRUSt (version 1.1.4; Langille et al. 2013), based on the Kyoto Encyclopedia of Genes and Genomes (KEGG) database.

### Molecular identification of the isolates by 16 S rDNA partial sequencing

Total genomic DNA from all morphotypes was extracted using the method described by Pitcher et al. (1989). The 16S rRNA gene was amplified using the universal primers 8F (5′-AGAGTTTGATCCTGGCTCAG-3′) (Lane, 1991) and 907R (5’-CCGTCAATTCMTTTRAGTTT-3’) (Muyzer et al. 1995). Amplification was performed using a touchdown PCR protocol, in which the annealing temperature was progressively reduced during the initial cycles to increase specificity and sequencing was carried out using an ABI Prism 3100 Genetic Analyzer (Applied Biosystems).

The resulting sequences were analyzed using Asparagin^®^ (Embrapa, Brazil), and consensus sequences (contigs) were assembled using BIOEDIT (version 7.0.5.3). Taxonomic identification was performed using the GenBank BLASTn tool. Identifications were assigned as follows: species-level for ≥ 97% identity, genus-level for 95–97% identity, and family/order-level for < 95% identity (Stackebrandt and Goebel 1994). The sequences were deposited in the GenBank library via the BankIt tool, with accession numbers available in Supplementary Table 1.

### Biofilm formation screening

The biofilm formation potential of the isolates was evaluated using an adapted protocol from Dias-Souza et al. (2013). Aliquots (200 µL) of overnight cultures of each strain in nutrient broth were dispensed in triplicate into untreated, sterile 96-well polystyrene microtiter plates. Wells without bacterial inoculum served as negative controls. Bacterial suspensions were standardized to an optical density (OD) of 0.5 at 600 nm (Varioskan, ThermoFisher, USA). The plates were incubated at 35 ± 2 °C overnight. After incubation, the wells were washed three times with 100 µL of sterile saline solution (0.85% NaCl) to remove non-adherent cells, and the remaining biofilm was resuspended in 190 µL of nutrient broth.

Biofilm cell viability was assessed by adding 10 µL of resazurin (0.1 g/) to each well, followed by incubation in the dark at 37 °C for 10 min. Resazurin is a blue dye that, when reduced by metabolically active bacteria to resorufin, turns pink and fluoresces (Pettit et al. 2009). Fluorescence was measured in arbitrary fluorescence units (AFU) using a Varioskan™ reader (λex 570 nm, λem 590 nm) (Gomes et al. 2013). Bacterial morphotypes were grouped based on AFU values using the k-means clustering algorithm in PAST software (version 1.90). Isolates with average fluorescence readings exceeding 1200 AFU were classified as adherent.

The ability of the bacteria to produce exopolysaccharides was also evaluated using Congo Red Agar (CRA), as described by Arciola et al. (2002). CRA plates were prepared by supplementing nutrient agar with 0.8% Congo red dye and 5% sucrose. After overnight incubation at 37 °C, biofilm-producing strains formed black colonies, while non-producers developed red to pink colonies. Colony colors were categorized using a reference scale: very black and black (normal slime producers) to very red, red, and pink (weak biofilm producers).

### Antimicrobial susceptibility test

Antimicrobial susceptibility was evaluated according to the Clinical and Laboratory Standards Institute (CLSI, 2022) guidelines. A total of 100 µL of 0.5 McFarland turbidity-adjusted overnight cultures were spread onto Mueller-Hinton agar plates (Difco, Becton Dickinson, USA). Antimicrobial disks (Sensifar, Brazil) containing 10 µg ampicillin, 30 µg cephalexin, 10 µg meropenem, 5 µg ciprofloxacin, 15 µg erythromycin, 30 µg chloramphenicol, 10 µg gentamicin, and 300 µg nitrofurantoin were placed on the plates, which were then incubated at 37 °C overnight. Inhibition zone diameters were measured after incubation. Standard strains of *Escherichia coli* ATCC 25,922, *Pseudomonas aeruginosa* ATCC 9027, and *Staphylococcus aureus* ATCC 6538 were used as quality controls.

Since reference susceptibility breakpoints were unavailable for most isolates, the inhibition zone diameters were grouped into low, intermediate, and high susceptibility categories (clusters 1, 2, and 3, respectively) using the k-means clustering algorithm. The inhibition zone size ranges for each cluster are detailed in Supplementary Table S3.

### Statistics

Data are presented as the arithmetic means ± (standard deviation of at least three independent experiments. Statistical analyses included analysis of variance (ANOVA), Fisher’s exact test, and chi-square tests. Interactions between biofilm age and structural factors were also evaluated. Pearson and likelihood correlation coefficients were calculated to identify potential associations between variables. A significance level of *p* < 0.05 was considered statistically significant. All statistical analyses were conducted using Minitab 17 for Windows.

Taxonomic classification and alpha and beta diversity analyses were performed using the core diversity pipeline of the QIIME package. Rarefaction curves were constructed based on the number of observed operational taxonomic units (OTUs) per read and evaluated using Good’s coverage analysis. Principal Coordinates Analysis (PCoA) was conducted using weighted UniFrac distance metrics. Bacterial diversity was estimated using the Shannon, Simpson, and Chao1 indices. Differential abundance of taxa was assessed using the edgeR package in R software.

## Results

### Operational parameters of the cooling towers

Most operational parameters remained similar between CTw 1 and CTw 2 (Tables 1 and 2), although some variations were observed throughout the experimental period. CTw 2 exhibited higher recirculation and evaporation rates (Table 1), which corresponds to its greater water capacity compared to CTw 1. Based on average values, CTw 2 showed higher bicarbonate alkalinity, total hardness, chlorides, residual-free chlorine, TSS, and sulfate concentrations (Table 2). In contrast, CTw 1 showed higher average values for concentration cycles, soluble phosphate, and soluble zinc, TOC, and NO_3_-N. However, maximum TSS and sulfate concentrations were higher in CTw 1 during specific sampling periods.

### THB counting, isolation, and identification

**Fig. 1:**
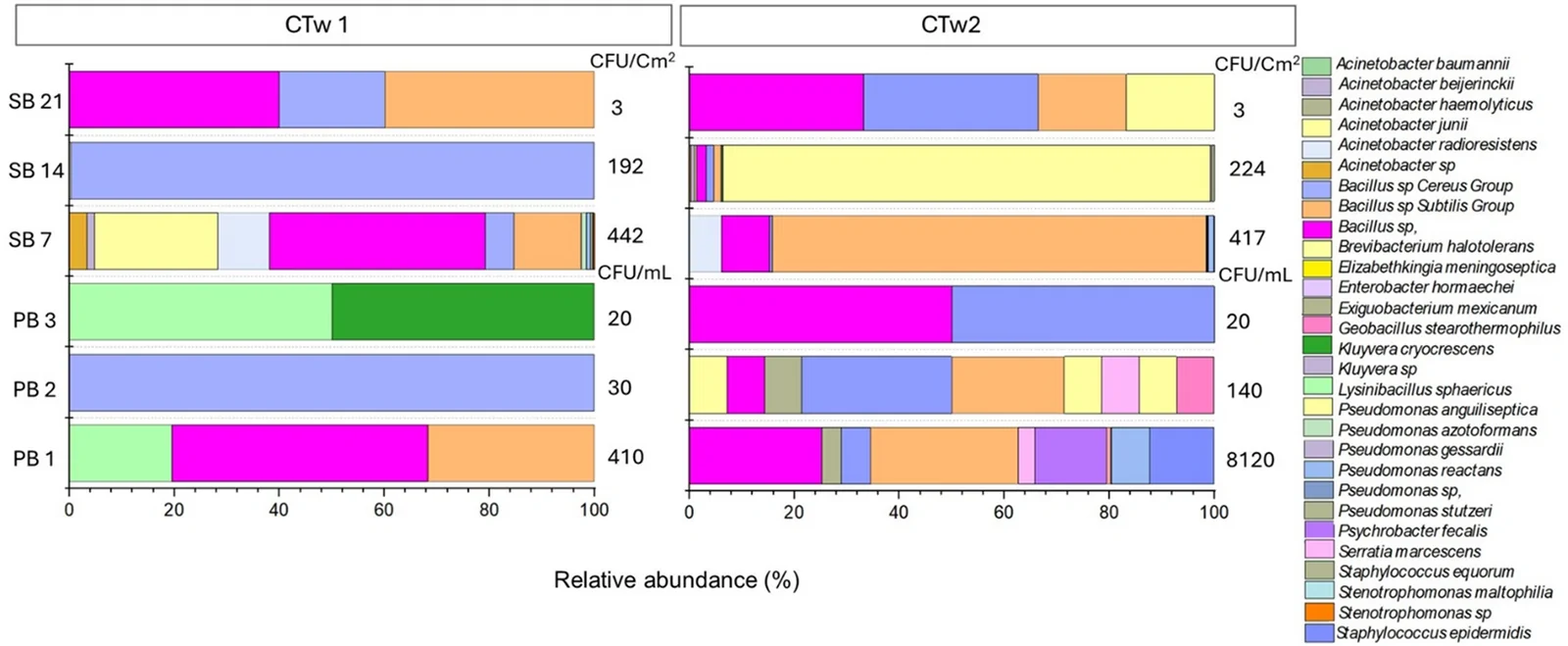
Barplots showing the relative abundance of bacterial species identified amidst the planktonic (PB) and sessile (SB) isolates in the CTw1 and CTw2 by culture-based approach. Numbers 7, 14 and 21 refer to the ages of the biofilms collectedin days. Number 1, 2 and 3 refer to the sampling

Planktonic (PB) and sessile (SB) bacterial densities were consistently higher in CTw 2 throughout the experimental period, although both towers showed fluctuations in total planktonic bacterial counts over time (Fig. 1). A total of 160 morphotypes were isolated: 54 from CTw 1 (45 sessile, 9 planktonic) and 106 from CTw 2 (70 sessile, 36 planktonic). The designation of planktonic bacteria refers to their sampling origin in the water column and does not imply inability to form biofilms. Based on 16 S rRNA gene sequencing, these isolates were classified into 14 genera and 22 species (Fig. 1). Partial 16 S rRNA gene sequences from these isolates have been deposited in GenBank under accession numbers KX856175 to KX856334 (Supplementary Table S2). In CTw 1, *Bacillus* was the most abundant planktonic genus at the beginning of the experiment (360 CFU/mL), followed by *Lysinibacillus* (90 CFU/mL) and *Kluyvera* (10 CFU/mL). *Bacillus* remained consistently present throughout the experimental period (Fig. 1). In contrast, CTw 2 exhibited a greater number of planktonic genera, with *Bacillus* (4890 CFU/mL) as the dominant genus, followed by *Acinetobacter* (320 CFU/mL), *Enterobacter* (260 CFU/mL), *Elizabethkingia* (10 CFU/mL), *Pseudomonas* (670 CFU/mL), *Staphylococcus* (1000 CFU/mL), Geobacillus (10 CFU/mL), *Psychrobacter* (1100 CFU/mL), and *Brevibacterium* (10 CFU/mL) (Fig. 1). Similarly, sessile isolates from CTw 2 exhibited a greater number of genera compared to CTw 1 (Fig. 1). *Bacillus* was the only genus consistently detected in biofilms throughout the experimental period in both CTw systems and was generally the most abundant across biofilms of different ages, except in 7-day-old biofilms from CTw 1, where *Acinetobacter* exhibited the highest density (363 CFU/cm²), and in 14-day-old biofilms from CTw 2, where *Elizabethkingia* reached the highest density (208 CFU/cm²).

Additionally, *Pseudomonas* (10 CFU/cm²) and *Stenotrophomonas* (0.92 CFU/cm²) were detected among sessile isolates from CTw 1 (Fig. 1). In CTw 2, *Acinetobacter* (29.6 CFU/cm²), *Exiguobacterium* (29.6 CFU/cm²), *Enterobacter* (0.52 CFU/cm²), *Pseudomonas* (4.52 CFU/cm²), *Serratia* (0.52 CFU/cm²), and *Staphylococcus* (1.03 CFU/cm²) were found. Interestingly, *Acinetobacter*, *Bacillus*, *Pseudomonas*, and *Stenotrophomonas* were detected in sessile samples from both towers.

### Taxonomic composition of the bacterial community in 7- and 14-day-old biofilms

The taxonomic composition of bacterial communities in biofilms formed on glass slides in both cooling towers (CTws) at 7 and 14 days was analyzed using 16 S rRNA high-throughput sequencing. To ensure data quality, singletons and operational taxonomic units (OTUs) unassigned at the phylum level were excluded from the dataset.

A total of 374,232.5 reads were obtained across all samples (± 16,464.63 standard deviation, SD), encompassing 2,151 OTUs (± 31 SD) (Table 3). Rarefaction curves reached a plateau, and Good’s Coverage values of 0.999 indicated a high sequencing depth, ensuring comprehensive microbial community characterization.

**Table 3:** Diversity analysis of biofilm-associated cells

Sample	Reads	OTUs *	Diversity index		
			Chao1	Shannon	Simpson
CTw 1/7 days	64,955 ± 11,961	458 ± 27	498.7 ± 17.6 d	4.92 ± 0.72 d	0.70 ± 0.06 d
CTw 2/ 7 days	99,695.5 ± 14,983.5	535 ± 48	573.05 ± 51.25 b	5.52 ± 0.55 b	0.94 ± 0.02 b
CTw 1/14days	115,864 ± 26,971	515 ± 27	550.7 ± 7.6 c	5.05 ± 0.98 c	0.88 ± 0.06 c
CTw 2/14days	93,718 ± 11,943	643 ± 22	691 ± 36.7 a	6.01 ± 0.27 a	0.95 ± 0.02 a

* Values represent mean ± standard deviation (SD). Total OTUs observed based on the number of reads. Numbers 7 and 14 indicate the biofilm age (days). Mean values followed by different lowercase letters differ significantly according to ANOVA followed by Fisher’s test (*p* < 0.05)

**Fig. 2:**
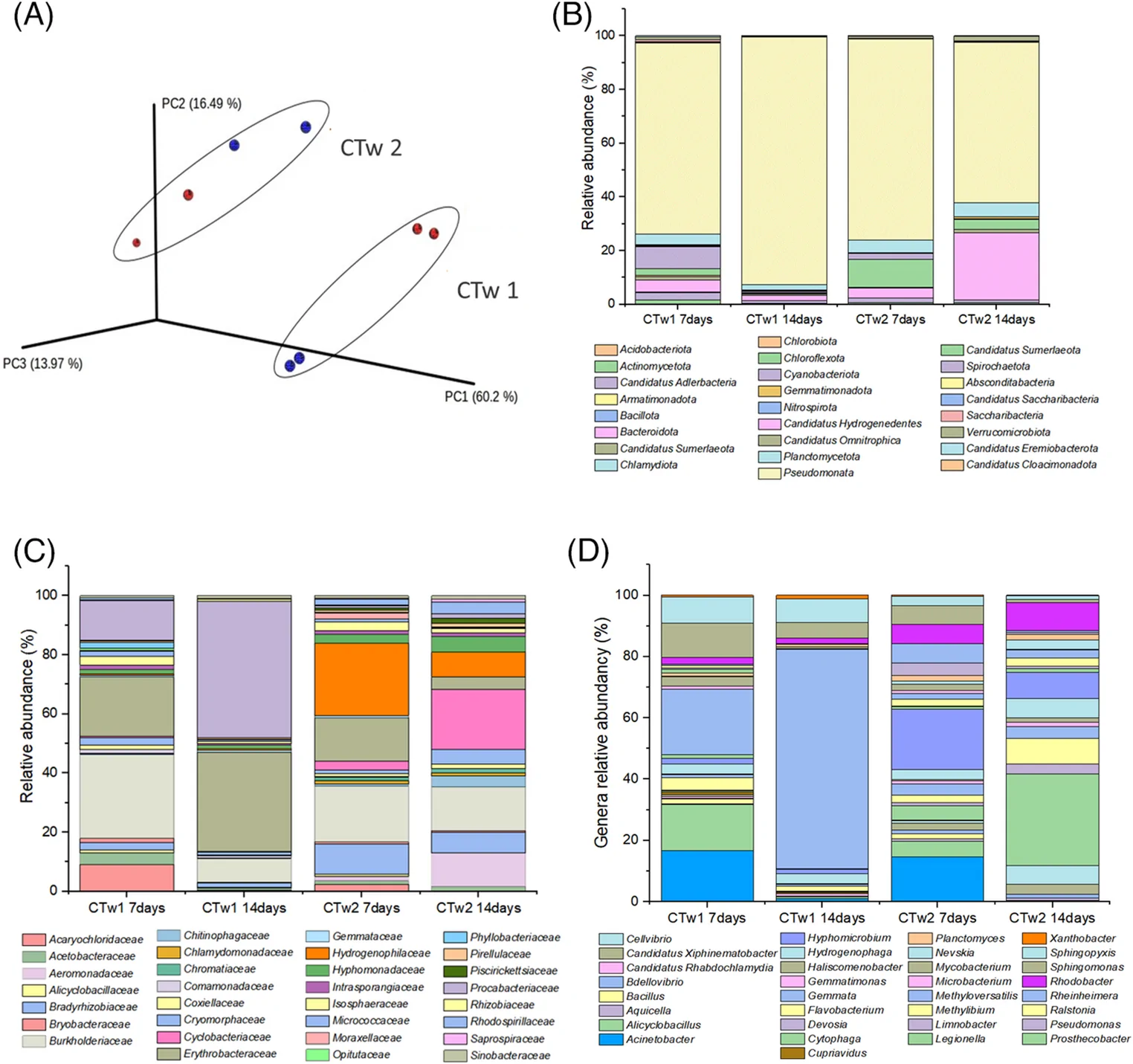
Principal Coordinate Analysis based on UNIFRAC weighted for the cooling towers (**A**). Relative abundance of phyla with RA > 0.1(**B**), family with RA > 0.5 (**C**) and genera with RA > 1 (**D**) on biofilms identified by metataxonomic approach. Blue dots: 7-day biofilms and Red dots: 14-day biofilms

Diversity estimators revealed higher values in CTw 2 overall, with increased diversity observed in 14-day-old biofilms regardless of the cooling tower (Table 3). Principal Coordinate Analysis (PCoA; Fig. 2A) showed clustering primarily by cooling tower, with additional separation based on biofilm age within each tower.

In CTw 1, Pseudomonadota was the most abundant phylum (73% on average), followed by Bacillota, Bacteroidota, and Cyanobacteriota (Fig. 2B). Over time, Pseudomonadota increased (7-day biofilms: 70%; 14-day biofilms: 91%), while Bacillota (7-day: 7.9%; 14-day: 0.65%) and Cyanobacteriota (7-day: 2.5%; 14-day: 0.2%) decreased (Fig. 2C). In CTw 2, Pseudomonadota remained dominant, with Bacteroidota, Cyanobacteriota, and Planctomycetota also present, and a significant reduction in Chlorobi was observed at 14 days (*p* < 0.05).

**Fig. 3:**
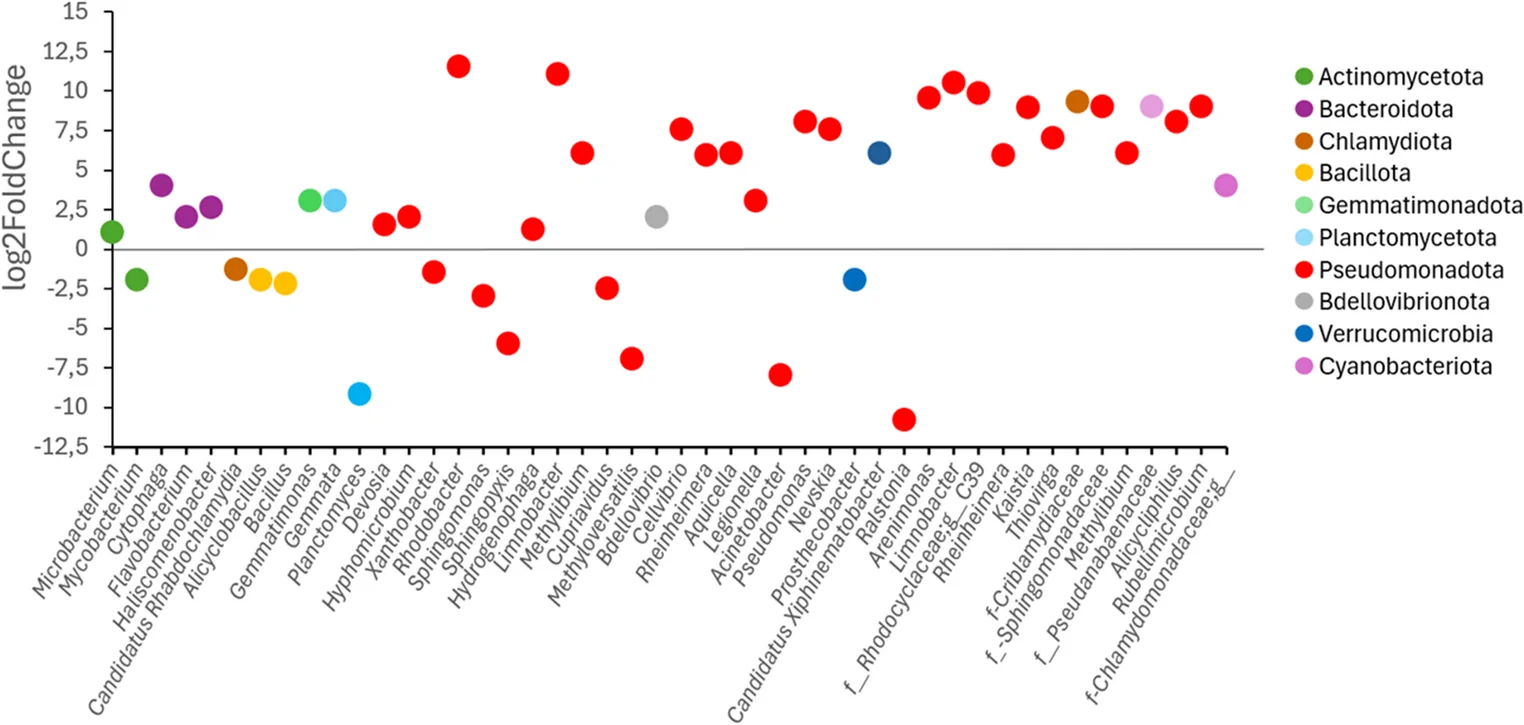
Differentially abundant bacterial genera in CTw1 identified using DESeq2 analysis

At the family level, *Procabacteriaceae*, *Erythrobacteriaceae*, and *Burkholderiaceae* were most abundant in CTw 1, along with *Acaryochloridaceae*, *Acetobacteraceae*, and *Bradyrhizobiaceae* (Fig. 2C). In contrast, *Burkholderiaceae*,* Hydrogenophilaceae*,* Cyclobacteriaceae*,* Erythrobacteraceae*,* Bradyrhizobiaceae*, and *Aeromonadaceae* predominated in CTw 2. At the genus level, the most abundant taxa in CTw 1 included *Methyloversatilis*, *Sphingopyxis*, *Acinetobacter*, *Alicyclobacillus*, and *Sphingomonas* (Fig. 2D). Between 7 and 14 days, *Methyloversatilis* (7 days: 9%; 14 days: 39.4%) and *Sphingopyxis* (7 days: 3.6%; 14 days: 4.3%) increased, whereas *Acinetobacter* (7 days: 7%; 14 days: 0.5%), *Alicyclobacillus* (7 days: 6.4%; 14 days: 0.4%), and *Sphingomonas* (7 days: 4.7%; 14 days: 2.7%) declined (*p* < 0.05) (Fig. 3). In CTw 2, the dominant genera were *Cytophaga*, *Hyphomicrobium*, *Rhodobacter*, *Flavobacterium*, and *Acinetobacter*, though no genera exhibited significant differences in abundance between 7- and 14-day-old biofilms (*p* > 0,05).

### PICRUSt functional prediction

**Fig. 4:**
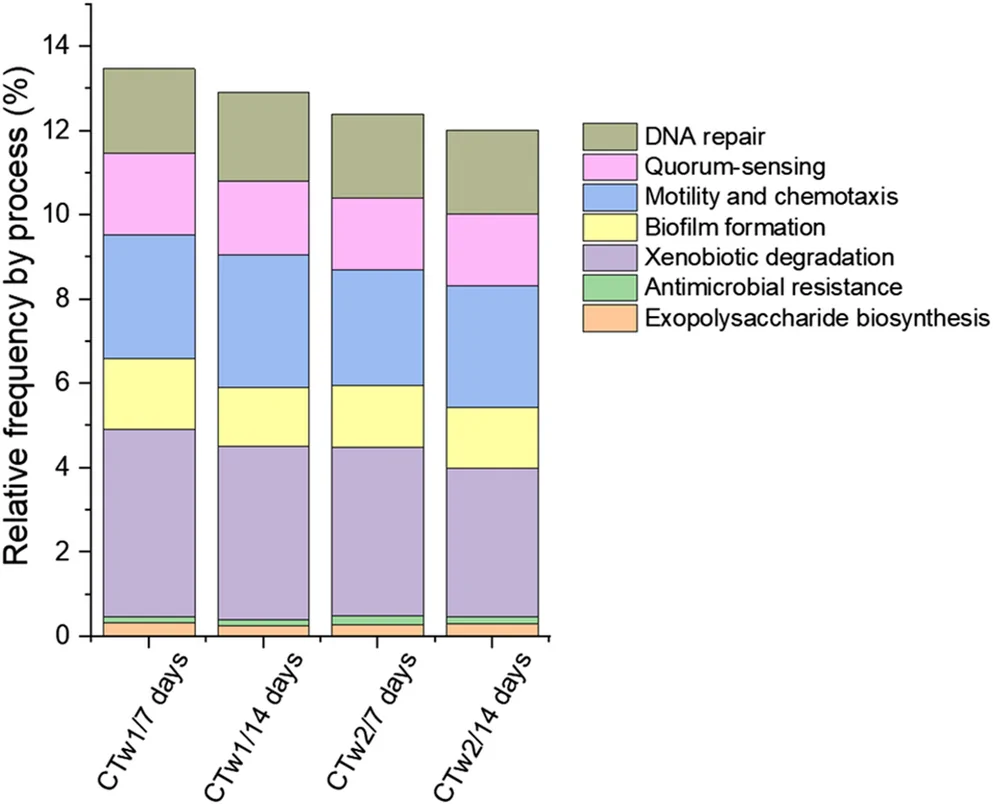
Barplots with the relative frequency of the predicted selected processes in each sample

Functional potential predictions using PICRUSt (Nearest Sequenced Taxon Index ≤ 0.139) identified 8,273 KEGG Orthologs (KOs). At the first functional level, six main categories were identified: metabolism (representing 50% of all KOs on average), genetic information processing (15.8%), environmental information processing (13.1%), cellular processes (4.8%), human diseases (1.3%), and organismal systems (0.8%). Unclassified KOs accounted for 14.2%, with minimal variation observed between biofilm ages or cooling towers. Key KOs associated with critical processes such as exopolysaccharide biosynthesis, antimicrobial resistance, xenobiotic degradation, biofilm formation, motility and chemotaxis, quorum-sensing, and DNA repair—processes critical to the studied system—were analyzed in detail (Fig. 4). A total of 447 KOs (12.6% on average, SD = 0.6%) was linked to these processes. The most abundant categories were xenobiotic degradation (4.1%, SD = 0.26), motility and chemotaxis (2.9%, SD = 0.15), and DNA repair (2%, SD = 0.05). Conversely, exopolysaccharide biosynthesis and antimicrobial resistance were less frequent, averaging 0.28% (SD = 0.03) and 0.16% (SD = 0.035), respectively.

### Biofilm formation assays

Biofilm production by all strains was evaluated using two methods: spotting cultures on CRA plates and cultivation in 96-well polystyrene plates. Among the 160 isolates, 34% (55 strains) formed black colonies, 52% (83 strains) formed red colonies, and 15% (22 strains) formed pink colonies. No additional color variations were observed (Supplementary Table S2).

K-means clustering analysis of biofilm formation in polystyrene plates is presented in Table 4. The type of cooling tower significantly influenced biofilm formation (*p* < 0.05), with higher values observed for bacteria from CTw1. Although biofilm formation tended to increase with biofilm age, this effect was not statistically significant (*p* > 0.05). Similarly, no significant differences in biofilm formation phenotypes were observed between sessile and planktonic isolates from either cooling tower. Bacteria classified as robust biofilm producers (UAF ≥ 1200, cluster 2) were predominantly from the genus *Bacillus*, whereas *Acinetobacter* isolates exhibited the lowest UAF values. The assays employed were intended to evaluate biofilm-forming phenotypes and adherence potential rather than detailed structural characterization of mature biofilms.

**Table 4:** Clusters of sessile and planktonic isolates according to biofilm forming potential in non-treated polystyrene plates

Group (AFU)	Number/percentage of bacteria on CTw						
	SB CTw 1 - Biofilm Age			SB CTw 2 - Biofilm Age			
	7 Days	14 days	21 days	7 Days	14 days	21 days	
1 (94–716.4)	8 (57%)	14 (51,5%)	1 (25%)	18 (69%)	22 (59.5%)	6 (86%)	
2 (1,228.6–2,829.1)	5 (36%)	8 (30%)	1 (25%)	7 (27%)	10 (27%)	1 (14%)	
3 (734-1,185.4)	1 (7%)	5 (18.5%)	2 (50%)	1 (4%)	5 (13.5%)	0	
Total	14 (100%)	27 (100%)	4 (100%)	26 (100%)	37 (100%)	7 (100%)	
Group (AFU)	PB - CTw 1			PB - CTw 2			
	1 (94–716.4)	2 (1185.4–2829.1)	3 (734–1228.6)	1 (94–716.4)	2 (1185.4–2829.1)	3 (734–1228.6)	
	3 (33,3%)	3 (33,3%)	3 (33,3%)	18 (51%)	15 (31%)	3 (18%)	

*SB* Sessible Bacteria. PB: Planktonic Bacteria, *CTw* Cooling tower, *AFU* Arbitrary fluorescence units. The type of CTw had effect on the biofilm formation pattern (*p* < 0.05)

### Antimicrobial susceptibility assays

**Fig. 5:**
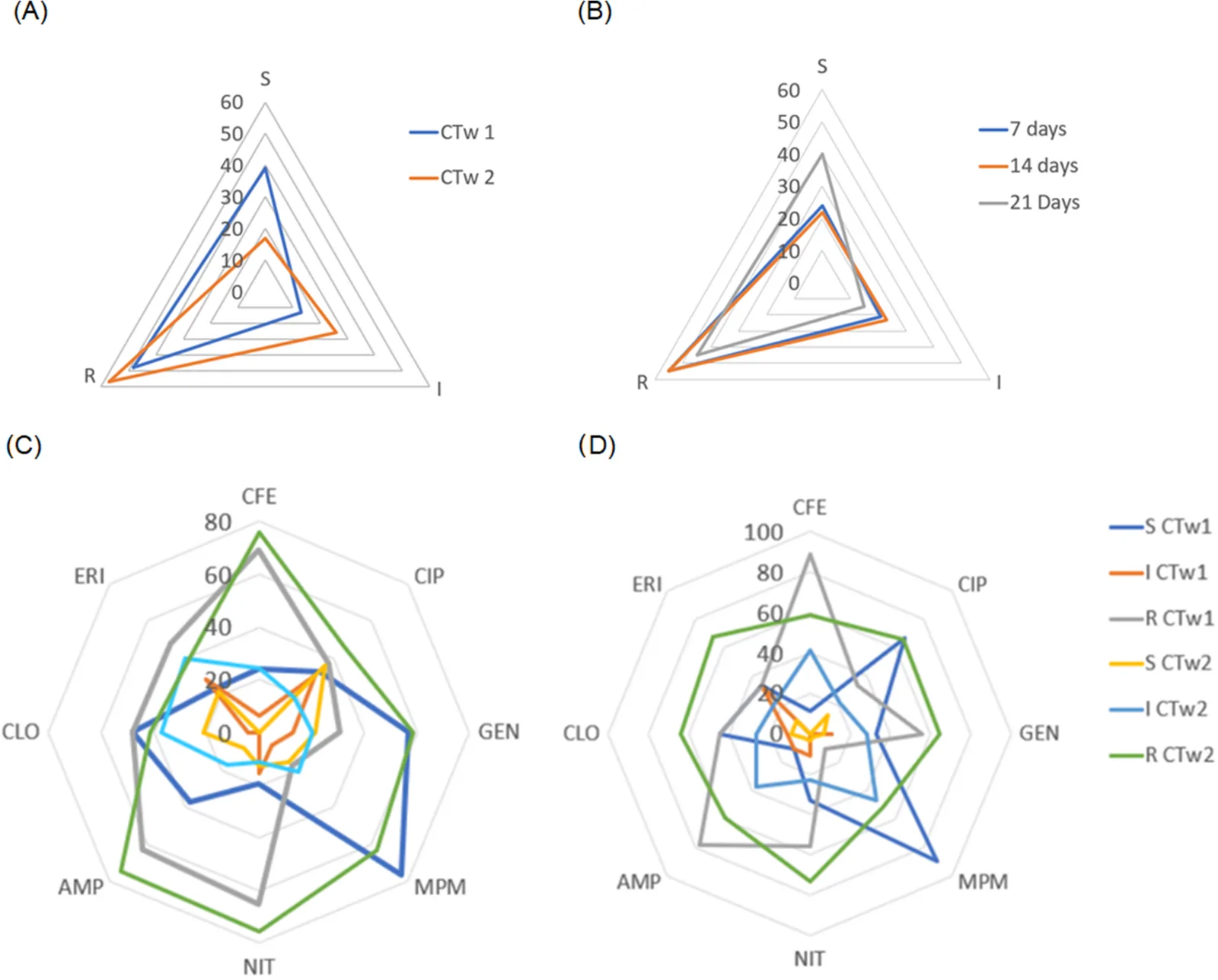
Global susceptibility profile of the 160 isolates to the tested antibiotics, expressed as percentage of bacteria classified as high susceptibility (S), Intermediate susceptibility (I) and poor susceptibility (R). (**A** and **C**) Planktonic and (**B** and **D**) Sessile bacteria. ERY: erythromycin; CLO: chloramphenicol; AMP: ampicilin; NIT: nitrofurantoin; MPM: meropenem; GEN: gentamycin; CIP: Ciprofloxacin; CFE: cephalexin

Antibiotics tested in this study were selected for their clinical relevance for treating infections caused by the species identified. Representative antibiotics from various chemical classes and modes of action were included. Cooling tower type significantly influenced susceptibility profiles, with isolates from CTw 2 exhibiting higher frequencies of reduced susceptibility compared to CTw 1 (*p* < 0.05). In CTw 1, 48% of isolates displayed low susceptibility, 13% showed intermediate susceptibility, and 39% exhibited high susceptibility (Fig. 5A). In contrast, in CTw 2, the distribution shifted to 57%, 26%, and 17%, respectively. Biofilm age also affected susceptibility patterns (*p* < 0.05), although no significant differences were observed between 7- and 14-day-old biofilms (Supplementary Table 3). However, 21-day-old biofilms demonstrated an increased frequency of isolates with intermediate or high susceptibility (*p* < 0.05) (Fig. 5B).

Overall, the resistance profile, based on the breakpoints established by CLSI, varied depending on the antimicrobial tested, regardless of the cooling tower or biofilm age. The highest susceptibility rates were observed with meropenem (55%), whereas cefalexin, ampicillin, and nitrofurantoin showed the highest rates of poor susceptibility (~ 70%). Gentamicin, ciprofloxacin, chloramphenicol, and erythromycin exhibited moderate rates of poor susceptibility (~ 50%) (Supplementary Table 3).

The antimicrobial resistance profile differed between planktonic and sessile isolates, as well as between the two cooling towers. For planktonic isolates, CTw1 exhibited higher susceptibility to the tested drugs, particularly meropenem, gentamicin, and chloramphenicol, compared to CTw2 (Fig. 5C). Although the resistance profiles were more similar between the two towers, sessile isolates from CTw1 demonstrated greater susceptibility to meropenem and ciprofloxacin (Fig. 5D). Moreover, sessile isolates from CTw1 exhibited lower overall resistance levels, with more pronounced differences between the two cooling towers.

## Discussion

In this study, THB densities cultured on Plate Count Agar (PCA) from CTw water samples were used to estimate microbial contamination levels. Results showed consistently higher densities of culturable planktonic and sessile bacteria in CTw 2 compared to CTw 1 (Fig. 1). Although culturable bacterial densities declined over time, metataxonomic analyses indicated that the overall bacterial populations did not decrease in either tower (Table 3). Specifically, in CTw 1, total reads and OTU number and the Chao1 richness estimator increased between 7 and 14 days, suggesting microbial growth or diversification. Similarly, in CTw 2, despite a slight reduction in total reads, OTUs, Chao1, and the Shannon diversity index also increased, reflecting gains in species richness and community evenness.

PCoA and taxonomic profiles (Fig. 2A, D) provided an independent view of community dynamics and confirmed the shifts suggested by cultivation-based analyses. The PCoA plot showed clear clustering by cooling tower, with additional separation between 7- and 14-day biofilms, indicating that both water source and biofilm age influenced microbial composition. CTw 1 biofilms showed decreased evenness over time, dominated by *Methyloversatilis* (approximately 40% relative abundance at 14 days), whereas it maintained richness comparable to 7 days. In CTw2, no single genus dominated; instead, the community displayed a more even distribution and increased richness over time. Importantly, some taxa were consistently detected across all sampling times in both towers, including *Bacillus* spp. and *Acinetobacter* spp., highlighting their resilience under different operational conditions. Other genera such as *Sphingopyxis* and *Pseudomonas* appeared intermittently, reflecting temporal variability.

Metataxonomic analysis also revealed a broad diversity of non-cultivable and difficult-to-cultivate bacterial taxa, including families such as *Burkholderiaceae*,* Comamonadaceae*, and *Sphingomonadaceae*, which are known for their roles in biofilm resilience and antimicrobial resistance (Vaz-Moreira et al. 2011; Alves Ruislan et al. 2024). These taxa were not recovered by culture-based analysis due to cultivation bias inherent to culture-dependent methods, as PCA selectively supports fast-growing, non-fastidious bacteria while failing to cultivate slow-growing or nutritionally demanding taxa (Amann et al. 1995; Vaz-Moreira et al. 2011).These findings indicate that physicochemical fluctuations—including total suspended solids (TSS), total organic carbon, oils and greases, soluble phosphate, total dissolved solids and chloride levels indicative of biocide application—influenced microbial community dynamics in both CTws. Furthermore, the open nature of cooling towers and environmental factors such as temperature, pH, rainfall and water flow likely contributed to these community fluctuations (Azam and Malfatti 2007). The coexistence of cultivable and non-cultivable bacteria with biofilm-forming capacity and antimicrobial resistance traits highlights the complexity of the CTw microbiome and underscores the value of combining culture-dependent and culture-independent approaches to accurately assess microbial diversity and potential health risks.

Despite total bacterial densities remaining below the microbiological thresholds recommended by the Cooling Towers Institute (CTI, 2008), which establishes planktonic bacterial counts below 10^4^ CFU/mL as acceptable for cooling tower systems, the detection of antibiotic-resistant species raises public health concerns. In the present study, planktonic bacterial densities remained below this limit throughout the experimental period. Cultivable analyses revealed that *Bacillus* (67%) was the predominant genus, followed by *Acinetobacter* (14%), along with *Serratia* and *Staphylococcus*. These bacteria, commonly found in soil and aquatic environments, are also implicated in human infections. *Acinetobacter* spp., for example, are frequently associated with sepsis and urinary tract infections, especially in immunocompromised individuals (Khawcharoenporn et al. 2014).

Other clinically relevant groups were also identified, reinforcing concerns about cooling towers as reservoirs of opportunistic pathogens. For instance, *Brevibacterium* spp., routinely isolated from soil, food and human skin, can act as opportunistic pathogens (Onraedt et al. 2005). *Elizabethkingia meningoseptica*, found in drinking water systems, is associated with multidrug-resistant meningitis (Ratnamani and Rao 2013). *Enterobacter hormaechei* has been associated with septic arthritis and urinary infections and is increasingly detected in water environments (Ben Said et al. 2015). *Staphylococcus* spp. are commonly present in water sources and linked to skin infections (Dias-Souza et al. 2013). *Serratia marcescens* is an opportunistic pathogen frequently isolated from urinary tract infections (Joyner et al. 2014), whereas *Kluyvera* spp., found in water, soil and sewage, can cause bacteremia (Altun et al. 2010) and *Stenotrophomonas maltophilia*, found in water treatment systems and sewage, is linked to respiratory and systemic infections (Brooke 2012). Notably, *Legionella* was detected in both towers by sequencing, which despite low overall bacterial counts can still represent an occupational health risk and is consistent with guidance and outbreak reports for cooling towers (Cooling Tower Institute 2008; REUTERS 2016).

Microbial diversity was lower in CTw 1 compared to CTw 2, likely due to differences in water sources and treatment processes. CTw 2 receives water from an artificial lagoon impacted by domestic and industrial effluents, treated by processes like clarification and chlorination, while CTw 1 relies on secondary effluent from the industry treated via EDR. The proximity of the towers and environmental factors such as wind speed and drag forces may facilitate microbial cross-contamination (Ruíz et al. 2013), explaining shared bacterial strains despite distinct inputs.

Several taxa identified by metataxonomic analysis are consistent with microbial communities reported in cooling tower systems, which are shaped by operational conditions, disinfectant exposure, and biofilm development (Türetgen 2004; Liu et al. 2009, 2011; Tsao et al. 2019; Alves Ruislan et al. 2024).In CTw 1, the dominance of Burkholderiaceae, together with the detection of Methyloversatilis, highlights the ecological role of members of Burkholderiales, a group frequently associated to pollutant degradation and adaptation to engineered water systems (Ju et al. 2015; Gunawardana et al. 2025). Similarly, members of the family Sphingomonadaceae has been reported in drinking-water systems and chlorinated environments, where some members show biofilm-forming capacity, persistence under disinfection pressure, and and resistance to antimicrobial compounds (Vaz-Moreira et al. 2011; Wang et al. 2014; Sun et al. 2013). Comamonadaceae, which was also detected in the present study, has requently been reported in polluted aquatic systems and activated sludge, reinforcing its ecological relevance in contaminated water environments and its potential role in organic pollutant degradation (Gunawardana et al. 2025; Cai et al. 2024).

Recent studies also highlight the presence of *Nitrospiraceae* and *Fusobacterium* in cooling tower microbiomes, suggesting roles in nutrient cycling and potential contributions to microbiologically influenced corrosion (Li et al. 2024; Zhu et al. 2025). Families such as Erythrobacteriaceae, Cytophagaceae, and Saprospiraceae are commonly associated with aquatic or biofilm-rich habitats and may contribute to organic matter turnover, whereas Cyclobacteriaceae comprises halotolerant taxa from marine or lake environments, suggesting ecological traits compatible with survival in cooling tower waters (Schwenk et al. 2014; Bhumika et al. 2013). Rhodocyclaceae, reported in hot springs and biogas-related substrates, further indicating ecological versatility across harsh environments (Badhai et al. 2015; Campanaro et al. 2016). Together, these findings confirm that the cooling tower microbiomes are enriched in bacterial groups with traits that may favor persistence, biofilm formation, and survival under nutrient-limited and disinfectant-exposed conditions in industrial water systems.

Genus-level analysis identified *Acinetobacter*,* Pseudomonas*, and *Bacillus* through both culture and sequencing methods, consistent with previous studies reporting these genera in cooling tower systems and their established roles in biofilm-associated corrosion, industrial biofouling, and opportunistic infections (Rajasekar et al. 2010; Hussain et al. 2013; Li et al. 2024). In contrast, the detection of genera such as *Methyloversatilis* and *Hyphomicrobium*, and *Sphingopyxis* expands the current knowledge of microbial diversity in industrial cooling tower systems, particularly when combining culture-dependent and high-throughput sequencing approaches. *Methyloversatilis* and *Hyphomicrobium* are typically associated with denitrification processes in aquatic ecosystems, but their presence in CTw systems remains undocumented. Other genera detected, such as *Sphingopyxis*, are commonly found in drinking water distribution systems, while genera like *Cytophaga*,* Rhodobacter*, and *Flavobacterium* have established roles in biofilm formation in cooling tower systems, further contributing to system fouling and maintenance challenges (Wang et al. 2013; Zhang et al. 2024; Alves Ruislan et al. 2024). Additionally, *Methylibium* and *Ralstonia* are often linked to corrosion in aquatic environments (van der Kooij et al. 2017; Foote et al. 2023). Finally, *Sphingomonas* species are noted for their resistance to chlorine-based treatments and ability to degrade micropollutants, suggesting they play a key role in biofilm resilience under antimicrobial and biocide pressure (Cydzik-Kwiatkowska and Zielińska 2016; Sun et al. 2013).

Concerning functional potential, the most prevalent biofilm-related KOs corresponded to *gspL*,* pilG*,* gcvA*,* wza*,* oxyR*,* csrA*,* gspE*,* gspD*, and *wza* genes. The first two, related to the type II secretion system and pili biogenesis (Korotkov et al. 2012; Chang 2018; Niu et al. 2020), suggest that these microbial communities have the potential to initiate attachment and biofilm formation through motility and surface adhesion, mechanisms well documented in biofilm-forming species such as *Pseudomonas*,* Acinetobacter*, and *Bacillus* (Liu et al. 2011; Hussain et al. 2013). Notably, the presence of the *wza* gene, which plays a key role in polysaccharide export and capsule formation, points to the ability of these communities to develop protective biofilm structures that enhance resistance to environmental stressors in industrial settings, such as cooling towers. This gene has been associated with the resilience of *Acinetobacter baumannii*, a common biofilm producer in clinical environments, but also found in industrial settings where it contributes to biofilm-associated corrosion. (Niu et al. 2020). Likewise, the genes *oxyR*, involved in oxidative stress response, and *csrA*, a global regulator of biofilm formation, suggest the capacity of these microbial communities to adapt to oxidative and chemical stresses commonly imposed by chlorination and industrial water treatment processes (Cui et al. 2025). These traits are frequently reported in taxa identified in the present study, including members of *Burkholderiaceae* and *Sphingomonadaceae*, which are recognized for their persistence in chlorinated water systems, xenobiotic degradation, and biofilm resilience (Vaz-Moreira et al. 2011; Sun et al. 2013). Furthermore, the *cysE* gene, linked to exopolysaccharide (EPS) biosynthesis, highlights the central role of EPS production in biofilm stability and protection, a hallmark of biofilm-forming bacteria such as *Bacillus*, and *Pseudomonas*, commonly involved in industrial biofouling and corrosion (Ivanova et al. 1999; Rajasekar et al. 2010; Alves Ruislan et al. 2024). Although PICRUSt provides predictive functional inference rather than direct confirmation of gene presence or expression, the predicted functional profiles were consistent with the dominant microbial groups identified in both cooling towers.

Antimicrobial susceptibility testing showed poor susceptibility to β-lactams and nitrofurantoin among most isolates, likely influenced by chlorine-based treatments and antimicrobial pollutants in the environment (Macauley et al. 2006; Mulamattathil et al. 2014). Resistance patterns were particularly pronounced in CTw 2, which receives water from a lagoon impacted by untreated sewage. Conventional water treatment systems struggle to remove micropollutants, raising concerns about water safety for reuse (Gomez et al. 2012). Prolonged exposure to low concentrations of antimicrobial agents fosters multidrug resistance, posing risks to both workers and surrounding communities. The observed high susceptibility to meropenem and poor susceptibility to other β-lactams aligns with expected patterns, with meropenem being structurally resistant to β-lactamase degradation (Nouda et al. 1992). Conversely, resistance to commonly prescribed antibiotics, such as cephalexin, ampicillin, and nitrofurantoin, may be due to environmental contamination with these drugs or their metabolites (Li et al. 2025; Zhu et al. 2025).

Few studies have evaluated the susceptibility of environmental *Bacillus* strains to antimicrobials. Our findings revealed a high frequency of strains with intermediate susceptibility to erythromycin and resistance to ciprofloxacin, nitrofurantoin, and β-lactams. This aligns with earlier studies reporting poor drug susceptibility among Bacillus species from marine environments and resistance to clindamycin, erythromycin and trimethoprim-sulfamethoxazole in terrestrial isolates (Ivanova et al. 1999; Luna et al. 2011). Similarly, Acinetobacter isolates exhibited poor susceptibility to chloramphenicol and ciprofloxacin, consistent with findings from wastewater treatment plants (Zhang et al. 2009). The persistence of antimicrobial-resistant bacteria in CTw systems highlights their potential as reservoirs for resistance genes that may disseminate via aerosols and effluent discharge, posing environmental and public health concerns (Zhang et al. 2009; Alves Ruislan et al. 2024; Zhu et al. 2025).

While microscopy-based approaches (e.g., confocal laser scanning microscopy, scanning electron microscopy) would provide detailed structural information on biofilm architecture, these analyses were beyond the scope of this screening study. Future work should incorporate such techniques to characterize biofilm organization and multispecies interactions.

## Conclusion

This study underscores the bacterial diversity, biofilm formation and antimicrobial susceptibility profiles in cooling towers. Although bacterial densities remained below corrosion risk thresholds, the presence of antibiotic-resistant and opportunistic pathogens presents significant public health concerns. Cooling towers may serve as reservoirs for these microorganisms, potentially contributing to localized endemicity. The higher diversity in towers receiving mixed effluents emphasizes the influence of water source and treatment processes on microbial community structure and dynamics. To mitigate these risks, enhanced water treatment processes, routine microbiological surveillance and antimicrobial susceptibility testing should be implemented as part of water quality management. Preventive measures to limit pathogen dissemination should be prioritized. As water quality management remains a global concern, safeguarding surface and groundwater from microbial contaminants is vital for public health and environmental stability.

## Supplementary Information

Below is the link to the electronic supplementary material.

Supplementary Material 1

## Data Availability

All data generated or analyzed during this study are included in this published article (in the tables). Additional information is available from the corresponding author upon reasonable request.

## Abbreviations

AFU: arbitrary fluorescence units
ARB: antibiotic-resistant bacteria
ARG: antimicrobial resistance genes
CFU: colony-forming units
CTw: cooling towers
EPS: extracellular polymeric substances
THB: total heterotrophic bacteria
