# Supplementary material for: Diversity, biofilm formation and antimicrobial susceptibility of aerobic heterotrophic bacteria isolated from cooling towers
Source: World J Microbiol Biotechnol. 2026 Jul 14;42(8):409. doi: 10.1007/s11274-026-05127-1 (PMC13368888; doi:10.1007/s11274-026-05127-1)
Supplement: Supplementary file 1 — Supplementary Material 1 [file 11274_2026_5127_MOESM1_ESM.docx]

Supplementary Tables and Figures

Table S1. Characteristics of the tested antimicrobials

| **Antibiotic** | **Concentration (µg/µL)** | **Mechanism of action** |
| --- | --- | --- |
| Amoxicillin + Clavulanate (AMC) | 30 | Interruption of peptidoglycan synthesis |
| Cephalothin (CFL) | 30 | Interruption of peptidoglycan synthesis |
| Ceftazidime (CAZ) | 30 | Interruption of peptidoglycan synthesis |
| Ciprofloxacin (CIP) | 5 | DNA gyrase inhibition |
| Imipenem (IMP) | 10 | Interruption of peptidoglycan synthesis |
| Sulfazotrim (SUT) | 25 | Inhibition of folic acid synthesis |
| Tetracycline (TET) | 30 | Inhibition of protein synthesis |

Supplementary Table 2 – Molecular identification of the bacteria isolated from the CTw. 2S – Sessile bacterium, tower 1. 2P – Planktonic bacterium, tower 1.

3S – Sessile bacterium, tower 2. 3P – Planktonic bacterium, tower 2.

| **Isolate Source/ Number** | | **Biofilm Age (Days)** | **Primary Identification** | **Accession Number** | **Coverage** | **Identity** | **Proposed Identification** | **Deposit Number** | **Congo-red**  **Agar** |
| --- | --- | --- | --- | --- | --- | --- | --- | --- | --- |
|  |  |  |  |  |  |  |  |  |  |
| 3S | 1 | 14 | *Bacillus* sp. | KJ410679 | 100% | 99% | *Bacillus* sp. | KX856175 | Red |
| 3S | 2 | 14 | *Acinetobacter beijerinckii* | KC152988 | 100% | 98% | *Acinetobacter beijerinckii* | KX856176 | Red |
| 3S | 3 | 14 | *Acinetobacter baumannii* | KM281496 | 100% | 99% | *Acinetobacter baumannii* | KX856177 | Red |
| 2S | 4 | 14 | *Bacillus cereus* | KM524118 | 100% | 99% | *Bacillus* sp. Cereus Group | KX856178 | Red |
| 3P | 5 | 14 | *Bacillus cereus* | KJ729602 | 100% | 100% | *Bacillus* sp. Cereus Group | KX856179 | Black |
| 3P | 6 | 14 | *Bacillus cereus* | KM524118 | 100% | 100% | *Bacillus* sp. Cereus Group | KX856180 | Red |
| 3S | 7 | 14 | *Bacillus cereus* | KM524118 | 100% | 100% | *Bacillus* sp. Cereus Group | KX856181 | Red |
| 3P | 8 | 14 | *Elizabethkingia meningoseptica* | FJ608007 | 100% | 99% | *Elizabethkingia meningoseptica* | KX856182 | Black |
| 3P | 9 | 14 | *Acinetobacter haemolyticus* | KJ009404 | 100% | 100% | *Acinetobacter haemolyticus* | KX856183 | Black |
| 3P | 10 | 14 | *Bacillus pumilus* | KF734912 | 100% | 100% | *Bacillus* sp. Subtilis Group | KX856184 | Red |
| 3S | 11 | 14 | *Bacillus anthracis* | KJ206080 | 100% | 100% | *Bacillus* sp. Cereus Group | KX856185 | Red |
| 3S | 12 | 14 | *Bacillus aryabhattai* | KJ534461 | 100% | 100% | *Bacillus* sp. | KX856186 | Black |
| 3S | 13 | 14 | *Acinetobacter beijerinckii* | JF742664 | 100% | 100% | *Acinetobacter beijerinckii* | KX856187 | Red |
| 3S | 14 | 14 | *Bacillus megaterium* | AB999847 | 100% | 99% | *Bacillus* sp. | KX856188 | Black |
| 3S | 15 | 14 | *Acinetobacter junii* | KM514310 | 100% | 99% | *Acinetobacter junii* | KX856189 | Red |
| 3S | 16 | 14 | *Bacillus safensis* | KM016940 | 100% | 100% | *Bacillus* sp. | KX856190 | Red |
| 2S | 17 | 21 | *Bacillus subtilis* | AM882680 | 100% | 97% | *Bacillus* sp. Subtilis Group | KX856191 | Red |
| 3S | 18 | 14 | *Bacillus safensis* | LN774188 | 100% | 99% | *Bacillus* sp. | KX856192 | Red |
| 3S | 19 | 14 | *Bacillus aryabhattai* | KJ573534 | 100% | 100% | *Bacillus* sp. | KX856193 | Red |
| 3P | 20 | 14 | *Acinetobacter junii* | LC014149 | 100% | 97% | *Acinetobacter junii* | KX856194 | Red |
| 3P | 21 | 14 | *Brevibacterium halotolerans* | KM817248 | 100% | 99% | *Brevibacterium halotolerans* | KX856195 | Red |
| 3S | 22 | 21 | *Bacillus cereus* | KJ729602 | 100% | 100% | *Bacillus* sp. Cereus Group | KX856196 | Red |
| 2S | 23 | 7 | *Bacillus cereus* | KF862926 | 100% | 100% | *Bacillus* sp. Cereus Group | KX856197 | Red |
| 3S | 24 | 14 | *Elizabethkingia meningoseptica* | EU128744 | 100% | 99% | *Elizabethkingia meningoseptica* | KX856198 | Red |
| 3S | 25 | 7 | *Bacillus* sp. | KJ410679 | 100% | 99% | *Bacillus* sp. | KX856199 | Black |
| 2P | 26 | 14 | *Bacillus cereus* | KJ612545 | 100% | 99% | *Bacillus* sp. Cereus Group | KX856200 | Red |
| 2P | 27 | 14 | *Bacillus cereus* | KJ612545 | 100% | 100% | *Bacillus* sp. Cereus Group | KX856201 | Red |
| 2S | 28 | 14 | *Bacillus cereus* | KJ612545 | 100% | 100% | *Bacillus* sp. Cereus Group | KX856202 | Red |
| 3S | 29 | 14 | *Acinetobacter* sp. | KC758138 | 100% | 99% | *Acinetobacter* sp. | KX856203 | Red |
| 3P | 30 | 7 | *Bacillus* sp*.* | JX514843 | 100% | 100% | *Bacillus* sp*.* | KX856204 | Black |
| 3S | 31 | 7 | *Bacillus* sp*.* | LN830896 | 100% | 99% | *Bacillus* sp*.* | KX856205 | Red |
| 3P | 32 | 7 | *Bacillus licheniformis* | KF879256 | 100% | 99% | *Bacillus* sp*.* Subtilis Group | KX856206 | Red |
| 3S | 33 | 7 | *Bacillus subtilis* | KM226922 | 100% | 99% | *Bacillus* sp*.* Subtilis Group | KX856207 | Red |
| 3S | 34 | 14 | *Bacillus anthracis* | KJ592610 | 87% | 100% | *Bacillus* sp*.* Cereus Group | KX856208 | Pink |
| 3P | 35 | 7 | *Pseudomonas azotoformans* | KR051236 | 100% | 99% | *Pseudomonas azotoformans* | KX856209 | Red |
| 3S | 36 | 7 | *Acinetobacter radioresistens* | KJ000864 | 100% | 98% | *Acinetobacter radioresistens* | KX856210 | Pink |
| 3P | 37 | 7 | *Bacillus amyloliquefaciens* | KP313602 | 98% | 99% | *Bacillus* sp*.* Subtilis Group | KX856211 | Red |
| 3S | 38 | 7 | *Bacillus anthracis* | KR019965 | 100% | 99% | *Bacillus* sp*.* Cereus Group | KX856212 | Pink |
| 3S | 39 | 7 | *Serratia marcescens* | KJ672369 | 100% | 100% | *Serratia marcescens* | KX856213 | Red |
| 2P | 40 | 21 | *Kluyvera cryocrescens* | AM933757 | 100% | 100% | *Kluyvera cryocrescens* | KX856214 | Black |
| 2S | 41 | 14 | *Acinetobacter junii* | HE651916 | 100% | 100% | *Acinetobacter junii* | KX856215 | Red |
| 3S | 42 | 14 | *Bacillus aryabhattai* | KF026344 | 100% | 99% | *Bacillus* sp*.* | KX856216 | Black |
| 2S | 43 | 21 | *Bacillus* sp. | KJ662651 | 100% | 99% | *Bacillus* sp*.* | KX856217 | Red |
| 3P | 44 | 14 | *Bacillus thuringiensis* | KF687039 | 100% | 99% | *Bacillus* sp*.* Cereus Group | KX856218 | Red |
| 3P | 45 | 14 | *Geobacillus stearothermophilus* | KM226938 | 100% | 98% | *Geobacillus stearothermophilus* | KX856219 | Pink |
| 3S | 46 | 14 | *Bacillus amyloliquefaciens* | CP003838 | 100% | 99% | *Bacillus* sp*.* Subtilis Group | KX856220 | Black |
| 2S | 47 | 21 | *Bacillus subtilis* | KJ682651 | 100% | 99% | *Bacillus* sp*.* Subtilis Group | KX856221 | Red |
| 3P | 48 | 14 | *Bacillus amyloliquefaciens* | KR063202 | 96% | 94% | *Bacillus* sp*.* Subtilis Group | KX856222 | Black |
| 3P | 49 | 14 | *Bacillus amyloliquefaciens* | KM453228 | 100% | 99% | *Bacillus* sp*.* Subtilis Group | KX856223 | Red |
| 2S | 50 | 14 | *Bacillus cereus* | KR006915 | 100% | 100% | *Bacillus* sp*.* Cereus Group | KX856224 | Red |
| 2S | 51 | 14 | *Bacillus amyloliquefaciens* | KR063193 | 100% | 99% | *Bacillus* sp*.* Subtilis Group | KX856225 | Black |
| 2S | 52 | 14 | *Pseudomonas gessardii* | KP761415 | 100% | 100% | *Pseudomonas gessardii* | KX856226 | Red |
| 2S | 53 | 14 | *Pseudomonas reactans* | KR051237 | 100% | 99% | *Pseudomonas reactans* | KX856227 | Red |
| 2S | 54 | 14 | *Bacillus circulans* | KF475811 | 100% | 99% | *Bacillus* sp*.* | KX856228 | Black |
| 2S | 55 | 14 | *Bacillus subtilis* | EU231615 | 100% | 99% | *Bacillus* sp*.* Subtilis Group | KX856229 | Black |
| 2S | 56 | 14 | *Acinetobacter radioresistens* | JX032809 | 100% | 99% | *Acinetobacter radioresistens* | KX856230 | Red |
| 3P | 57 | 14 | *Pseudomonas anguilliseptica* | KF261005 | 100% | 100% | *Pseudomonas anguilliseptica* | KX856231 | Pink |
| 3P | 58 | 14 | *Bacillus circulans* | JN644554 | 100% | 99% | *Bacillus* sp*.* | KX856232 | Black |
| 3S | 59 | 14 | *Bacillus amyloliquefaciens* | KP209386 | 100% | 100% | *Bacillus* sp*.* Subtilis Group | KX856233 | Red |
| 2P | 60 | 7 | *Bacillus* sp. | LN774742 | 100% | 99% | *Bacillus* sp. | KX856234 | Black |
| 3S | 61 | 7 | *Bacillus pumilus* | HQ388809 | 97% | 96% | *Bacillus* sp*.* Subtilis Group | KX856235 | Red |
| 3S | 62 | 7 | *Stenotrophomonas* sp. | KJ534283 | 100% | 93% | *Stenotrophomonas* sp*.* | KX856236 | Red |
| 3S | 63 | 7 | *Exiguobacterium mexicanum* | KJ722476 | 100% | 100% | *Exiguobacterium mexicanum* | KX856237 | Black |
| 2P | 64 | 7 | *Lysinibacillus sphaericus* | KF151165 | 100% | 99% | *Lysinibacillus sphaericus* | KX856238 | Pink |
| 2S | 65 | 7 | *Acinetobacter radioresistens* | HG917871 | 97% | 90% | *Acinetobacter* sp*.* | KX856239 | Red |
| 3P | 66 | 7 | *Bacillus thuringiensis* | KF956588 | 100% | 100% | *Bacillus* sp*.* Cereus Group | KX856240 | Red |
| 3S | 67 | 7 | *Acinetobacter radioresistens* | KJ018232 | 100% | 99% | *Acinetobacter radioresistens* | KX856241 | Red |
| 2S | 68 | 7 | *Acinetobacter radioresistens* | KF150371 | 100% | 100% | *Acinetobacter radioresistens* | KX856242 | Red |
| 3P | 69 | 7 | *Pseudomonas* sp. | HF571536 | 100% | 100% | *Pseudomonas* sp*.* | KX856243 | Red |
| 3P | 70 | 7 | *Bacillus thuringiensis* | KP004438 | 100% | 100% | *Bacillus* sp*.* Cereus Group | KX856244 | Red |
| 3P | 71 | 7 | *Bacillus* sp*.* | KP265905 | 100% | 100% | *Bacillus* sp*.* | KX856245 | Red |
| 3P | 72 | 7 | *Staphylococcus epidermidis* | KM099438 | 100% | 100% | *Staphylococcus epidermidis* | KX856246 | Black |
| 2S | 73 | 7 | *Bacillus cereus* | KM524118 | 100% | 99% | *Bacillus* sp*.* Cereus Group | KX856247 | Red |
| 2S | 74 | 7 | *Bacillus thuringiensis* | KP004438 | 100% | 100% | *Bacillus* sp*.* Cereus Group | KX856248 | Pink |
| 2P | 75 | 7 | *Bacillus amyloliquefaciens* | KJ528275 | 96% | 94% | *Bacillus* sp*.* Subtilis Group | KX856249 | Black |
| 3P | 76 | 7 | *Bacillus cereus* | KP298707 | 100% | 99% | *Bacillus* sp*.* Cereus Group | KX856250 | Black |
| 3S | 77 | 7 | *Bacillus pumilus* | KJ528879 | 96% | 99% | *Bacillus* sp*.* Subtilis Group | KX856251 | Red |
| 2S | 78 | 7 | *Acinetobacter junii* | KJ788686 | 100% | 99% | *Acinetobacter junii* | KX856252 | Red |
| 3S | 79 | 14 | *Elizabethkingia meningoseptica* | JX473586 | 82% | 97% | *Elizabethkingia meningoseptica* | KX856253 | Red |
| 3S | 80 | 14 | *Acinetobacter junii* | KJ147085 | 100% | 98% | *Acinetobacter junii* | KX856254 | Red |
| 3S | 81 | 14 | *Staphylococcus equorum* | KJ531646 | 100% | 100% | *Staphylococcus equorum* | KX856255 | Black |
| 3S | 82 | 14 | *Staphylococcus equorum* | KM036089 | 100% | 98% | *Staphylococcus equorum* | KX856256 | Red |
| 3S | 83 | 14 | *Bacillus* sp. | KJ410679 | 100% | 100% | *Bacillus* sp*.* | KX856257 | Red |
| 3S | 84 | 7 | *Bacillus thuringiensis* | KF971833 | 100% | 99% | *Bacillus* sp*.* Cereus Group | KX856258 | Black |
| 2S | 85 | 14 | *Bacillus amyloliquefaciens* | CP007244 | 98% | 99% | *Bacillus* sp*.* Subtilis Group | KX856259 | Black |
| 3S | 86 | 7 | *Bacillus cereus* | KM009129 | 100% | 100% | *Bacillus* sp*.* Cereus Group | KX856260 | Red |
| 2S | 87 | 14 | *Stenotrophomonas* sp. | EF221778 | 97% | 85% | *Stenotrophomonas* sp*.* | KX856261 | Red |
| 2S | 88 | 14 | *Bacillus amyloliquefaciens* | GU250447 | 98% | 99% | *Bacillus* sp*.* Subtilis Group | KX856262 | Red |
| 3S | 89 | 7 | *Bacillus methylotrophicus* | CP011347 | 100% | 99% | *Bacillus* sp*.* | KX856263 | Black |
| 3S | 90 | 7 | *Bacillus amyloliquefaciens* | KP219718 | 100% | 99% | *Bacillus* sp*.* Subtilis Group | KX856264 | Black |
| 3S | 91 | 21 | *Bacillus aryabhattai* | KF026336 | 100% | 100% | *Bacillus* sp*.* | KX856265 | Red |
| 2S | 92 | 14 | *Pseudomonas azotoformans* | KP720568 | 100% | 99% | *Pseudomonas azotoformans* | KX856266 | Red |
| 3P | 93 | 7 | *Bacillus pumilus* | LC034563 | 97% | 96% | *Bacillus* sp*.* Subtilis Group | KX856267 | Red |
| 2S | 94 | 14 | *Stenotrophomonas maltophilia* | JX392997 | 100% | 99% | *Stenotrophomonas maltophilia* | KX856268 | Red |
| 2S | 95 | 14 | *Bacillus cereus* | AB996598 | 100% | 100% | *Bacillus* sp*.* Cereus Group | KX856269 | Red |
| 2P | 96 | 7 | *Bacillus pumilus* | LC034563 | 100% | 99% | *Bacillus* sp*.* Subtilis Group | KX856270 | Black |
| 2S | 97 | 14 | *Acinetobacter junii* | KP257784 | 100% | 99% | *Acinetobacter junii* | KX856271 | Black |
| 3S | 98 | 7 | *Bacillus cereus* | KR063194 | 100% | 99% | *Bacillus* sp*.* Cereus Group | KX856272 | Black |
| 3S | 99 | 14 | *Acinetobacter beijerinckii* | KM219997 | 100% | 99% | *Acinetobacter beijerinckii* | KX856273 | Red |
| 3S | 100 | 7 | *Bacillus cereus* | HQ670580 | 100% | 99% | *Bacillus* sp*.* Cereus Group | KX856274 | Pink |
| 2S | 101 | 14 | *Bacillus cereus* | KJ398239 | 100% | 99% | *Bacillus* sp*.* Cereus Group | KX856275 | Black |
| 3S | 102 | 7 | *Bacillus circulans* | KF599078 | 100% | 100% | *Bacillus* sp*.* | KX856276 | Black |
| 3S | 103 | 7 | *Bacillus pumilus* | HM854261 | 100% | 96% | *Bacillus* sp*.* Subtilis Group | KX856277 | Pink |
| 2S | 104 | 7 | *Acinetobacter radioresitens* | KF150371 | 100% | 100% | *Acinetobacter radioresitens* | KX856278 | Red |
| 3S | 105 | 7 | *Bacillus pumilus* | KJ528879 | 99% | 99% | *Bacillus* sp*.* Subtilis Group | KX856279 | Red |
| 3S | 106 | 7 | *Bacillus safensis* | KM016940 | 100% | 100% | *Bacillus* sp*.* | KX856280 | Black |
| 2S | 107 | 7 | *Acinetobacter beijerinckii* | KJ009404 | 100% | 99% | *Acinetobacter beijerinckii* | KX856281 | Red |
| 3P | 108 | 7 | *Enterobacter hormaechei* | KJ742536 | 100% | 99% | *Enterobacter hormaechei* | KX856282 | Black |
| 3S | 109 | 7 | *Pseudomonas libanensis* | AB741818 | 100% | 94% | *Pseudomonas* sp*.* | KX856283 | Black |
| 3S | 110 | 7 | *Bacillus* sp*.* | KP296559 | 100% | 100% | *Bacillus* sp*.* | KX856284 | Red |
| 2S | 111 | 14 | *Pseudomonas stutzeri* | KJ580521 | 100% | 100% | *Pseudomonas stutzeri* | KX856285 | Red |
| 3S | 112 | 14 | *Bacillus aryabhattai* | KF026337 | 100% | 100% | *Bacillus* sp*.* | KX856286 | Black |
| 2S | 113 | 14 | *Bacillus anthracis* | KJ591023 | 100% | 99% | *Bacillus* sp*.* Cereus Group | KX856287 | Black |
| 2S | 114 | 14 | *Bacillus amyloliquefaciens* | KP219718 | 99% | 100% | *Bacillus* sp*.* Subtilis Group | KX856288 | Black |
| 2S | 115 | 21 | *Bacillus cereus* | KR006917 | 100% | 99% | *Bacillus* sp*.* Cereus Group | KX856289 | Pink |
| 2P | 116 | 21 | *Lysinibacillus* sp*haericus* | KJ184908 | 100% | 99% | *Lysinibacillus sphaericus* | KX856290 | Red |
| 3S | 117 | 21 | *Bacillus cereus* | CP009686 | 100% | 97% | *Bacillus* sp*.* Cereus Group | KX856291 | Black |
| 3S | 118 | 14 | *Acinetobacter haemolyticus* | KJ395367 | 100% | 99% | *Acinetobacter haemolyticus* | KX856292 | Black |
| 2S | 119 | 21 | *Bacillus megaterium* | LC005453 | 100% | 99% | *Bacillus* sp*.* | KX856293 | Black |
| 3S | 120 | 21 | *Bacillus megaterium* | KP230453 | 100% | 100% | *Bacillus* sp*.* | KX856294 | Black |
| 2S | 121 | 14 | *Acinetobacter junii* | KM281504 | 100% | 99% | *Acinetobacter junii* | KX856295 | Black |
| 2S | 122 | 14 | *Bacillus cereus* | KF241553 | 91% | 95% | *Bacillus* sp*.* Cereus Group | KX856296 | Pink |
| 3S | 123 | 14 | *Enterobacter hormaechei* | KJ742536 | 100% | 99% | *Enterobacter hormaechei* | KX856297 | Black |
| 3S | 124 | 14 | *Bacillus cereus* | KP720573 | 99% | 99% | *Bacillus* sp*.* Cereus Group | KX856298 | Black |
| 3P | 125 | 7 | *Acinetobacter haemolyticus* | KJ395367 | 100% | 99% | *Acinetobacter haemolyticus* | KX856299 | Red |
| 2S | 126 | 14 | *Bacillus thuringiensis* | KP004438 | 100% | 99% | *Bacillus* sp*.* Cereus Group | KX856300 | Red |
| 3S | 127 | 14 | *Bacillus licheniformis* | KF879261 | 100% | 99% | *Bacillus* sp*.* Subtilis Group | KX856301 | Black |
| 3S | 128 | 7 | *Kluyvera* sp. | KJ880014 | 100% | 95% | *Kluyvera* sp*.* | KX856302 | Pink |
| 3S | 129 | 21 | *Pseudomonas anguilliseptica* | HM103328 | 100% | 100% | *Pseudomonas anguilliseptica* | KX856303 | Pink |
| 2S | 130 | 7 | *Bacillus amyloliquefaciens* | KP690978 | 100% | 99% | *Bacillus* sp*.* Subtilis Group | KX856304 | Pink |
| 2S | 131 | 7 | *Bacillus subtilis* | FJ803233 | 100% | 99% | *Bacillus* sp*.* Subtilis Group | KX856305 | Black |
| 2S | 132 | 7 | *Bacillus subtilis* | KJ592619.2 | 100% | 99% | *Bacillus* sp*.* Subtilis Group | KX856306 | Pink |
| 2S | 133 | 7 | *Bacillus amyloliquefaciens* | GU250447 | 100% | 99% | *Bacillus* sp*.* Subtilis Group | KX856307 | Black |
| 2P | 134 | 14 | *Bacillus anthracis* | CP009328 | 100% | 99% | *Bacillus* sp*.* Cereus Group | KX856308 | Red |
| 3P | 135 | 7 | *Bacillus amyloliquefaciens* | CP011278 | 100% | 100% | *Bacillus* sp*.* Subtilis Group | KX856309 | Pink |
| 3P | 136 | 7 | *Bacillus amyloliquefaciens* | KJ528275 | 100% | 99% | *Bacillus* sp*.* Subtilis Group | KX856310 | Black |
| 3P | 137 | 14 | *Bacillus thuringiensis* | CP009600 | 95% | 98% | *Bacillus* sp*.* Cereus Group | KX856311 | Pink |
| 2S | 138 | 7 | *Bacillus anthracis* | KP261079 | 100% | 97% | *Bacillus* sp*.* Cereus Group | KX856312 | Red |
| 3S | 139 | 7 | *Bacillus aryabhattai* | KP284271 | 91% | 100% | *Bacillus* sp*.* | KX856313 | Pink |
| 3S | 140 | 14 | *Bacillus subtilis* | AM882680 | 100% | 98% | *Bacillus* sp*.* Subtilis Group | KX856314 | Red |
| 3P | 141 | 7 | *Bacillus pumilus* | LC034563 | 100% | 99% | *Bacillus* sp*.* Subtilis Group | KX856315 | Red |
| 3P | 142 | 14 | *Bacillus pumilus* | LC034563 | 100% | 99% | *Bacillus* sp*.* Subtilis Group | KX856316 | Pink |
| 3S | 143 | 14 | *Bacillus aryabhattai* | KF026344 | 100% | 99% | *Bacillus* sp*.* | KX856317 | Black |
| 3S | 144 | 14 | *Bacillus subtilis* | KP273194 | 100% | 99% | *Bacillus* sp*.* Subtilis Group | KX856318 | Red |
| 3S | 145 | 7 | *Bacillus thuringiensis* | GQ421197 | 95% | 99% | *Bacillus* sp*.* Cereus Group | KX856319 | Pink |
| 3S | 146 | 21 | *Bacillus pumilus* | GQ280098 | 100% | 98% | *Bacillus* sp*.* Subtilis Group | KX856320 | Red |
| 3S | 147 | 14 | *Bacillus amyloliquefaciens* | CP002634 | 100% | 99% | *Bacillus* sp*.* Subtilis Group | KX856321 | Red |
| 3S | 148 | 14 | *Bacillus amyloliquefaciens* | KM453228 | 100% | 99% | *Bacillus* sp*.* Subtilis Group | KX856322 | Red |
| 3P | 149 | 7 | *Psychrobacter fecalis* | LK391545 | 100% | 99% | *Psychrobacter fecalis* | KX856323 | Black |
| 2S | 150 | 14 | *Bacillus cereus* | DQ420176 | 99% | 99% | *Bacillus* sp*.* Cereus Group | KX856324 | Black |
| 3P | 151 | 14 | *Bacillus safensis* | LN774188 | 99% | 98% | *Bacillus* sp*.* | KX856325 | Black |
| 3P | 152 | 14 | *Enterobacter hormaechei* | KC431781 | 100% | 99% | *Enterobacter hormaechei* | KX856326 | Red |
| 2S | 153 | 14 | *Bacillus* sp*.* | EF061441 | 100% | 98% | *Bacillus* sp*.* | KX856327 | Pink |
| 3S | 154 | 21 | *Bacillus circulans* | KF599078 | 100% | 99% | *Bacillus* sp*.* | KX856328 | Black |
| 3S | 155 | 14 | *Bacillus circulans* | KC441853 | 100% | 99% | *Bacillus* sp*.* | KX856329 | Black |
| 3S | 156 | 14 | *Bacillus cereus* | KP765746 | 100% | 98% | *Bacillus* sp*.* Cereus Group | KX856330 | Black |
| 3P | 157 | 21 | *Bacillus thuringiensis* | KF150502 | 100% | 99% | *Bacillus* sp*.* Cereus Group | KX856331 | Red |
| 2S | 158 | 14 | *Bacillus cereus* | HF570102 | 99% | 96% | *Bacillus* sp*.* Cereus Group | KX856332 | Black |
| 3S | 159 | 14 | *Elizabethkingia meningoseptica* | JX067927 | 100% | 99% | *Elizabethkingia meningoseptica* | KX856333 | Red |
| 2S | 160 | 7 | *Bacillus cereus* | HQ694049 | 100% | 99% | *Bacillus* sp*.* Cereus Group | KX856334 | Red |

Supplementary table 3 – Antimicrobial susceptibility of the CTw isolates. Size ranges (in mm) were established for each antimicrobial disk using the *K-means* clustering algorithm of the software PAST (version 1.90). G1 – 1: Group 1, tower 1. G2 – 1: Group 2, tower 1. G3 – 1: Group 3, tower 1. G1 – 2: Group 1, tower 2. G2 – 2: Group 2, tower 2. G3 – 2: Group 3, tower 2.

| **Cefalexin** | | | |  | **Ciprofloxacin** | | | |  | **Gentamicin** | | | |  | **Meropenem** | | | |
| --- | --- | --- | --- | --- | --- | --- | --- | --- | --- | --- | --- | --- | --- | --- | --- | --- | --- | --- |
| **Inhibition Zone Group** | **Age (Days)** | **Bacterial Origin** | **Total of Isolates** |  | **Inhibition Zone Group** | **Age (Days)** | **Bacterial Origin** | **Total of Isolates** |  | **Inhibition Zone Group** | **Age (Days)** | **Bacterial Origin** | **Total of Isolates** |  | **Inhibition Zone Group** | **Age (Days)** | **Bacterial Origin** | **Total of Isolates** |
| G1 – 1 (no zone inhibition) | 7 | Sessile | 5 |  | G1 - 1 (0 - 17 mm) | 7 | Sessile | 5 |  | G1 - 1 (0 - 9 mm) | 7 | Sessile | 2 |  | G1 - 1 (12 - 22 mm) | 7 | Sessile | 5 |
|  |  | Planktonic | 3 |  |  |  | Planktonic | 0 |  |  |  | Planktonic | 0 |  |  |  | Planktonic | 2 |
|  | 14 | Sessile | 6 |  |  | 14 | Sessile | 11 |  |  | 14 | Sessile | 0 |  |  | 14 | Sessile | 9 |
|  |  | Planktonic | 0 |  |  |  | Planktonic | 2 |  |  |  | Planktonic | 0 |  |  |  | Planktonic | 3 |
|  | 21 | Sessile | 2 |  |  | 21 | Sessile | 1 |  |  | 21 | Sessile | 0 |  |  | 21 | Sessile | 1 |
|  |  | Planktonic | 0 |  |  |  | Planktonic | 0 |  |  |  | Planktonic | 0 |  |  |  | Planktonic | 1 |
| G2 - 1 (8 - 18 mm) | 7 | Sessile | 6 |  | G2 - 1 (18 - 25 mm) | 7 | Sessile | 0 |  | G2 - 1 (10 - 17 mm) | 7 | Sessile | 4 |  | G2 - 1 (23 - 28 mm) | 7 | Sessile | 6 |
|  |  | Planktonic | 1 |  |  |  | Planktonic | 1 |  |  |  | Planktonic | 2 |  |  |  | Planktonic | 1 |
|  | 14 | Sessile | 14 |  |  | 14 | Sessile | 1 |  |  | 14 | Sessile | 16 |  |  | 14 | Sessile | 7 |
|  |  | Planktonic | 4 |  |  |  | Planktonic | 0 |  |  |  | Planktonic | 3 |  |  |  | Planktonic | 0 |
|  | 21 | Sessile | 5 |  |  | 21 | Sessile | 0 |  |  | 21 | Sessile | 3 |  |  | 21 | Sessile | 3 |
|  |  | Planktonic | 0 |  |  |  | Planktonic | 0 |  |  |  | Planktonic | 0 |  |  |  | Planktonic | 0 |
| G3 - 1 (19 - 36 mm) | 7 | Sessile | 3 |  | G3 - 1 (26 - 33 mm) | 7 | Sessile | 9 |  | G3 - 1 (18 - 30 mm) | 7 | Sessile | 7 |  | G3 - 1 (29 - 37 mm) | 7 | Sessile | 3 |
|  |  | Planktonic | 0 |  |  |  | Planktonic | 3 |  |  |  | Planktonic | 2 |  |  |  | Planktonic | 1 |
|  | 14 | Sessile | 5 |  |  | 14 | Sessile | 15 |  |  | 14 | Sessile | 11 |  |  | 14 | Sessile | 11 |
|  |  | Planktonic | 1 |  |  |  | Planktonic | 1 |  |  |  | Planktonic | 0 |  |  |  | Planktonic | 0 |
|  | 21 | Sessile | 0 |  |  | 21 | Sessile | 5 |  |  | 21 | Sessile | 2 |  |  | 21 | Sessile | 1 |
|  |  | Planktonic | 0 |  |  |  | Planktonic | 2 |  |  |  | Planktonic | 2 |  |  |  | Planktonic | 1 |
| **Nitrofurantoin** | | | |  | **Ampicilin** | | | |  | **Chloramphenicol** | | | |  | **Erythromycin** | | | |
| G1 - 1 (0 - 14 mm) | 7 | Sessile | 12 |  | G1 - 1 (0 - 17 mm) | 7 | Sessile | 14 |  | G1 - 1 (0 - 9 mm) | 7 | Sessile | 5 |  | G1 - 1 (12 - 22 mm) | 7 | Sessile | 2 |
|  |  | Planktonic | 2 |  |  |  | Planktonic | 5 |  |  |  | Planktonic | 2 |  |  |  | Planktonic | 0 |
|  | 14 | Sessile | 22 |  |  | 14 | Sessile | 14 |  |  | 14 | Sessile | 13 |  |  | 14 | Sessile | 3 |
|  |  | Planktonic | 2 |  |  |  | Planktonic | 0 |  |  |  | Planktonic | 2 |  |  |  | Planktonic | 1 |
|  | 21 | Sessile | 5 |  |  | 21 | Sessile | 3 |  |  | 21 | Sessile | 3 |  |  | 21 | Sessile | 2 |
|  |  | Planktonic | 1 |  |  |  | Planktonic | 1 |  |  |  | Planktonic | 0 |  |  |  | Planktonic | 0 |
| G2 - 1 (15 - 19 mm) | 7 | Sessile | 1 |  | G2 - 1 (18 - 25 mm) | 7 | Sessile | 0 |  | G2 - 1 (10 - 17 mm) | 7 | Sessile | 4 |  | G2 - 1 (23 - 28 mm) | 7 | Sessile | 8 |
|  |  | Planktonic | 2 |  |  |  | Planktonic | 0 |  |  |  | Planktonic | 0 |  |  |  | Planktonic | 5 |
|  | 14 | Sessile | 5 |  |  | 14 | Sessile | 5 |  |  | 14 | Sessile | 4 |  |  | 14 | Sessile | 12 |
|  |  | Planktonic | 1 |  |  |  | Planktonic | 1 |  |  |  | Planktonic | 0 |  |  |  | Planktonic | 1 |
|  | 21 | Sessile | 0 |  |  | 21 | Sessile | 3 |  |  | 21 | Sessile | 0 |  |  | 21 | Sessile | 2 |
|  |  | Planktonic | 0 |  |  |  | Planktonic | 1 |  |  |  | Planktonic | 0 |  |  |  | Planktonic | 2 |
| G3 - 1 (20 - 38 mm) | 7 | Sessile | 0 |  | G3 - 1 (26 - 33 mm) | 7 | Sessile | 1 |  | G3 - 1 (18 - 30 mm) | 7 | Sessile | 6 |  | G3 - 1 (29 - 37 mm) | 7 | Sessile | 5 |
|  |  | Planktonic | 0 |  |  |  | Planktonic | 0 |  |  |  | Planktonic | 3 |  |  |  | Planktonic | 0 |
|  | 14 | Sessile | 0 |  |  | 14 | Sessile | 4 |  |  | 14 | Sessile | 8 |  |  | 14 | Sessile | 10 |
|  |  | Planktonic | 0 |  |  |  | Planktonic | 1 |  |  |  | Planktonic | 0 |  |  |  | Planktonic | 0 |
|  | 21 | Sessile | 0 |  |  | 21 | Sessile | 0 |  |  | 21 | Sessile | 3 |  |  | 21 | Sessile | 2 |
|  |  | Planktonic | 1 |  |  |  | Planktonic | 0 |  |  |  | Planktonic | 2 |  |  |  | Planktonic | 0 |
| **Cefalexin** | | | |  | **Ciprofloxacin** | | | |  | **Gentamicin** | | | |  | **Meropenem** | | | |
| G1 - 2 (no Zone inhibition) | 7 | Sessile | 9 |  | G1 - 2 (0 - 17 mm) | 7 | Sessile | 9 |  | G1 - 2 (0 - 9 mm) | 7 | Sessile | 3 |  | G1 - 2 (12 - 22 mm) | 7 | Sessile | 14 |
|  |  | Planktonic | 6 |  |  |  | Planktonic | 2 |  |  |  | Planktonic | 0 |  |  |  | Planktonic | 4 |
|  | 14 | Sessile | 10 |  |  | 14 | Sessile | 14 |  |  | 14 | Sessile | 0 |  |  | 14 | Sessile | 17 |
|  |  | Planktonic | 7 |  |  |  | Planktonic | 6 |  |  |  | Planktonic | 1 |  |  |  | Planktonic | 5 |
|  | 21 | Sessile | 2 |  |  | 21 | Sessile | 4 |  |  | 21 | Sessile | 0 |  |  | 21 | Sessile | 1 |
|  |  | Planktonic | 1 |  |  |  | Planktonic | 1 |  |  |  | Planktonic | 0 |  |  |  | Planktonic | 0 |
| G2 - 2 (8 - 18 mm) | 7 | Sessile | 8 |  | G2 - 2 (18 - 25 mm) | 7 | Sessile | 3 |  | G2 - 2 (10 - 17 mm) | 7 | Sessile | 11 |  | G2 - 2 (23 - 28 mm) | 7 | Sessile | 5 |
|  |  | Planktonic | 3 |  |  |  | Planktonic | 5 |  |  |  | Planktonic | 7 |  |  |  | Planktonic | 7 |
|  | 14 | Sessile | 13 |  |  | 14 | Sessile | 4 |  |  | 14 | Sessile | 11 |  |  | 14 | Sessile | 14 |
|  |  | Planktonic | 10 |  |  |  | Planktonic | 4 |  |  |  | Planktonic | 6 |  |  |  | Planktonic | 4 |
|  | 21 | Sessile | 4 |  |  | 21 | Sessile | 1 |  |  | 21 | Sessile | 4 |  |  | 21 | Sessile | 2 |
|  |  | Planktonic | 0 |  |  |  | Planktonic | 0 |  |  |  | Planktonic | 0 |  |  |  | Planktonic | 1 |
| G3 - 2 (19 - 36 mm) | 7 | Sessile | 10 |  | G3 - 2 (26 - 33 mm) | 7 | Sessile | 14 |  | G3 - 2 (18 - 30 mm) | 7 | Sessile | 12 |  | G3 - 2 (29 - 37 mm) | 7 | Sessile | 7 |
|  |  | Planktonic | 4 |  |  |  | Planktonic | 10 |  |  |  | Planktonic | 10 |  |  |  | Planktonic | 5 |
|  | 14 | Sessile | 13 |  |  | 14 | Sessile | 19 |  |  | 14 | Sessile | 26 |  |  | 14 | Sessile | 6 |
|  |  | Planktonic | 3 |  |  |  | Planktonic | 7 |  |  |  | Planktonic | 10 |  |  |  | Planktonic | 8 |
|  | 21 | Sessile | 1 |  |  | 21 | Sessile | 2 |  |  | 21 | Sessile | 3 |  |  | 21 | Sessile | 3 |
|  |  | Planktonic | 0 |  |  |  | Planktonic | 0 |  |  |  | Planktonic | 1 |  |  |  | Planktonic | 0 |
| **Nitrofurantoin** | | | |  | **Ampicilin** | | | |  | **Chloramphenicol** | | | |  | **Erythromycin** | | | |
| G1 - 2 (0 - 14 mm) | 7 | Sessile | 20 |  | G1 - 2 (0 - 17 mm) | 7 | Sessile | 17 |  | G1 - 2 (0 - 9 mm) | 7 | Sessile | 16 |  | G1 - 2 (12 - 22 mm) | 7 | Sessile | 3 |
|  |  | Planktonic | 13 |  |  |  | Planktonic | 9 |  |  |  | Planktonic | 7 |  |  |  | Planktonic | 4 |
|  | 14 | Sessile | 32 |  |  | 14 | Sessile | 19 |  |  | 14 | Sessile | 12 |  |  | 14 | Sessile | 13 |
|  |  | Planktonic | 14 |  |  |  | Planktonic | 13 |  |  |  | Planktonic | 5 |  |  |  | Planktonic | 0 |
|  | 21 | Sessile | 7 |  |  | 21 | Sessile | 3 |  |  | 21 | Sessile | 3 |  |  | 21 | Sessile | 0 |
|  |  | Planktonic | 1 |  |  |  | Planktonic | 0 |  |  |  | Planktonic | 0 |  |  |  | Planktonic | 0 |
| G2 - 2 (15 - 19 mm) | 7 | Sessile | 6 |  | G2 - 2 (18 - 25 mm) | 7 | Sessile | 4 |  | G2 - 2 (10 - 17 mm) | 7 | Sessile | 5 |  | G2 - 2 (23 - 28 mm) | 7 | Sessile | 13 |
|  |  | Planktonic | 4 |  |  |  | Planktonic | 5 |  |  |  | Planktonic | 3 |  |  |  | Planktonic | 9 |
|  | 14 | Sessile | 3 |  |  | 14 | Sessile | 5 |  |  | 14 | Sessile | 11 |  |  | 14 | Sessile | 12 |
|  |  | Planktonic | 3 |  |  |  | Planktonic | 4 |  |  |  | Planktonic | 8 |  |  |  | Planktonic | 13 |
|  | 21 | Sessile | 0 |  |  | 21 | Sessile | 0 |  |  | 21 | Sessile | 2 |  |  | 21 | Sessile | 4 |
|  |  | Planktonic | 0 |  |  |  | Planktonic | 0 |  |  |  | Planktonic | 0 |  |  |  | Planktonic | 0 |
| G3 - 2 (20 - 38 mm) | 7 | Sessile | 1 |  | G3 - 2 (26 - 33 mm) | 7 | Sessile | 6 |  | G3 - 2 (18 - 30 mm) | 7 | Sessile | 6 |  | G3 - 2 (29 - 37 mm) | 7 | Sessile | 11 |
|  |  | Planktonic | 0 |  |  |  | Planktonic | 1 |  |  |  | Planktonic | 5 |  |  |  | Planktonic | 2 |
|  | 14 | Sessile | 2 |  |  | 14 | Sessile | 12 |  |  | 14 | Sessile | 14 |  |  | 14 | Sessile | 10 |
|  |  | Planktonic | 0 |  |  |  | Planktonic | 3 |  |  |  | Planktonic | 7 |  |  |  | Planktonic | 7 |
|  | 21 | Sessile | 0 |  |  | 21 | Sessile | 4 |  |  | 21 | Sessile | 2 |  |  | 21 | Sessile | 3 |
|  |  | Planktonic | 0 |  |  |  | Planktonic | 0 |  |  |  | Planktonic | 0 |  |  |  | Planktonic | 0 |
